# Supplementary material for: Studies of prevalence: how a basic epidemiology concept has gained recognition in the COVID-19 pandemic
Source: BMJ Open. 2022 Oct 27;12(10):e061497. doi: 10.1136/bmjopen-2022-061497 (PMC9620521; doi:10.1136/bmjopen-2022-061497)
Supplement: Author's manuscript [file bmjopen-2022-061497.draft_revisions.pdf]

BMJ Open is committed to open peer review. As part of this commitment we make the peer review history of every article we publish publicly available.

When an article is published we post the peer reviewers' comments and the authors' responses online. We also post the versions of the paper that were used during peer review. These are the versions that the peer review comments apply to.

The versions of the paper that follow are the versions that were submitted during the peer review process. They are not the versions of record or the final published versions. They should not be cited or distributed as the published version of this manuscript.

BMJ Open is an open access journal and the full, final, typeset and author-corrected version of record of the manuscript is available on our site with no access controls, subscription charges or pay-per-view fees (http://bmjopen.bmj.com).

If you have any questions on BMJ Open's open peer review process please email info.bmjopen@bmj.com

BMJ Open

The science of prevalence. How a basic epidemiology concept has gained relevance in the COVID-19 pandemic.

Journal:	*BMJ Open*
Manuscript ID	bmjopen-2022-061497.R1
Article Type:	Communication
Date Submitted by the Author:	22-Apr-2022
Complete List of Authors:	Buitrago-Garcia, Diana; University of Bern Faculty of Medicine, Institute of Social and Preventive Medicine (ISPM); University of Bern, Graduate School of Health Sciences<br>Salanti, Georgia; University of Bern Faculty of Medicine, Institute of Social and Preventive Medicine<br>Low, Nicola; University of Bern Faculty of Medicine, Institute of Social and Preventive Medicine
**Primary Subject Heading**:	Epidemiology
Secondary Subject Heading:	Infectious diseases
Keywords:	EPIDEMIOLOGY, COVID-19, STATISTICS & RESEARCH METHODS

SCHOLARONE™
Manuscripts

I, the Submitting Author has the right to grant and does grant on behalf of all authors of the Work (as defined in the below author licence), an exclusive licence and/or a non-exclusive licence for contributions from authors who are: i) UK Crown employees; ii) where BMJ has agreed a CC-BY licence shall apply, and/or iii) in accordance with the terms applicable for US Federal Government officers or employees acting as part of their official duties; on a worldwide, perpetual, irrevocable, royalty-free basis to BMJ Publishing Group Ltd ("BMJ") its licensees and where the relevant Journal is co-owned by BMJ to the co-owners of the Journal, to publish the Work in BMJ Open and any other BMJ products and to exploit all rights, as set out in our licence.

The Submitting Author accepts and understands that any supply made under these terms is made by BMJ to the Submitting Author unless you are acting as an employee on behalf of your employer or a postgraduate student of an affiliated institution which is paying any applicable article publishing charge ("APC") for Open Access articles. Where the Submitting Author wishes to make the Work available on an Open Access basis (and intends to pay the relevant APC), the terms of reuse of such Open Access shall be governed by a Creative Commons licence – details of these licences and which Creative Commons licence will apply to this Work are set out in our licence referred to above.

Other than as permitted in any relevant BMJ Author's Self Archiving Policies, I confirm this Work has not been accepted for publication elsewhere, is not being considered for publication elsewhere and does not duplicate material already published. I confirm all authors consent to publication of this Work and authorise the granting of this licence.

For peer review only

Communication article

The science of prevalence. How a basic epidemiology concept has gained relevance in the COVID-19 pandemic

Diana Buitrago-Garcia

Institute of Social and Preventive Medicine, Graduate School of Health Sciences, University of Bern, Bern, Switzerland (0000-0001-9761-206X)

Email: diana.buitragogarcia@ispm.unibe.ch

Georgia Salanti

Institute of Social and Preventive Medicine, University of Bern, Bern, Switzerland (0000-0002-3830-8508)

Email: georgia.salanti@ispm.unibe.ch

Nicola Low

Institute of Social and Preventive Medicine, University of Bern, Bern, Switzerland (0000-0003-4817-8986)

Email: nicola.low@ispm.unibe.ch

Correspondence: Nicola Low, Professor of Epidemiology and Public Health, Institute of Social and Preventive Medicine, University of Bern, 43 Mittelstrasse, Bern, CH-3012, Switzerland. Email: nicola.low@ispm.unibe.ch

Keywords

Epidemiology, COVID-19, Statistic and research methods

Main text 2628 words, 1 figure

For peer review only

Abstract

Prevalence measures the occurrence of any health condition, exposure, or other factors related to health. The experience of COVID-19, a new disease caused by severe acute respiratory syndrome coronavirus 2 (SARS-CoV-2), has highlighted the importance of prevalence studies, for which issues of reporting and methodology have traditionally been neglected. This communication highlights key issues about risks of bias in the design and conduct of prevalence studies and in reporting them, using examples about SARS-CoV-2 and COVID-19. The two main domains of bias in prevalence studies are those related to the study population (selection bias) and the condition or risk factor being assessed (information biases). Sources of selection bias should be considered both at the time of the invitation to take part in a study and when assessing who participates and provides valid data (respondents and non-respondents). Information bias appears when there are systematic errors affecting the accuracy and reproducibility of the measurement of the condition or risk factor. Types of information bias include misclassification, observer and recall bias. When reporting prevalence studies, clear descriptions of the target population, study population, study setting and context, and clear definitions of the condition or risk factor and its measurement, are essential. Without clear reporting, the risks of bias cannot be assessed properly. Bias in the findings of prevalence studies can, however, impact decision-making and the spread of disease. The concepts and examples provided here can be applied to any condition or outcome assessed through prevalence. Efforts to strengthen methodological research about, and improve the quality of, studies of prevalence in all fields of research should continue beyond this pandemic.

For peer review only

In introductory epidemiology, students learn about prevalence, an easy to understand concept, defined as "*a proportion that measures disease occurrence of any type of health condition, exposure, or other factor related to health*",[1] or "*the proportion of persons in a population who have a particular disease or attribute at a specified point in time or over a specified period.*"[2] Prevalence is an important measure for assessing the magnitude of health-related conditions, and studies of prevalence are an important source of information for estimating the burden of disease, injuries and risk factors.[3] Accurate information about prevalence enables health authorities to assess the health needs of a population, to develop prevention programmes and prioritise resources to improve public health.[4] Perhaps, owing to the apparent simplicity of the concept of prevalence, methodological developments to assess the quality of reporting, the potential for bias, and the synthesis of prevalence estimates in meta-analysis have been neglected,[5] when compared with the attention paid to methods relevant to evidence from randomised controlled trials and comparative observational studies.[6 7]

The COVID-19 pandemic has shown the need for epidemiological studies to describe and understand a new disease quickly but accurately.[8] Studies reporting on prevalence have been an important source of evidence to describe the prevalence of active SARS-CoV-2 infection and antibodies to SARS-CoV-2, the spectrum of SARS-CoV-2-related morbidity and helped to understand factors related to infection and disease to inform national decisions about containment measures.[9-11] Accurate estimates of prevalence of SARS-CoV-2 are also crucial because they are used as an input for the estimation of other quantities, such as infection fatality ratios, which can be calculated indirectly using seroprevalence estimates.[12] Assessments of published studies have, however, highlighted methodological issues that affect study design, conduct, analysis, interpretation, and reporting.[13-15]

The objective of this communication is to highlight key issues about the risk of bias in studies that measure prevalence and about the quality of reporting, using examples about SARS-CoV-2 and COVID-19. Although incidence and prevalence are related epidemiologically, we do not discuss incidence in this article because the study designs for measurement of the quantities differ. Bias is a systematic deviation of results or inferences from the underlying (unobserved) true values.[1] The risk of bias is a judgment about the degree to which the methods or findings of a study might under-or over-

For peer review only

estimate the true value in the target population,[7] in this case, the prevalence of a condition or risk factor. Quality of reporting refers to the completeness and transparency of the presentation of a research publication.[16] Risk of bias and quality of reporting are separate, but closely related, because it is only possible to assess the strengths and weaknesses of a study report if the methods and results are described adequately.

Bias in prevalence studies

The two main domains of bias in prevalence studies are those related to the study population (selection bias) and the condition being assessed (information biases) (Fig. 1). Biases involved in the design, conduct and analysis of a study affect its internal validity.[1] Selection bias also affects external validity, the extent to which findings from a specific study can be generalised to a wider, target population in time and space.[1] There are many names given to different biases, often addressing the same concept. For this communication, we use the names and definitions published in the Dictionary of Epidemiology.[1]

Selection bias

Selection bias relates to the representativeness of the sample used to estimate the prevalence in relation to the target population. The target population is the group of individuals to whom the findings, conclusions, or inferences from a study can be generalised.[1] There are two steps in a prevalence study at which selection bias might occur: at the invitation to take part in the study and, among those invited, who take part (Fig. 1).

[Fig 1.]

Selection bias in the invitation to take part in the study: the probability of being invited to take part in a study should be the same for every person in the target population. Those who are invited to take part might be defined by demographic characteristics, e.g., children below ten years or study setting (e.g., hospitalised patients), or a random sample of the general population. The least biased method to select participants in a prevalence study is to sample at random from the target population. For example, the Real-time Assessment of Community Transmission (REACT) Studies to assess the

For peer review only

prevalence of the virus, using molecular diagnostic tests (REACT-1) and antibodies (REACT-2), invite random samples of people, stratified by area, from the National Health Service patient list in England.[9] Those invited are close to a truly random sample because almost everyone in England is registered with a general practitioner. Some of the processes involved in the generation of a random sample might still result in bias. For example, underestimation of SARS-CoV-2 seroprevalence in older adults could occur if the sampling frame excludes people in long term care facilities, who have been at higher risk of exposure than the average in the general population.[13] Other methods of enrolment are at risk of selection bias if those invited differ systematically from the target population and those differences are associated with the condition of interest. Asking for volunteers through advertisements are liable to selection bias because not everyone has the same probability of seeing or replying to the advert. For example, the use of social media to invite people to a drive-in test centre to estimate the population prevalence of antibodies to SARS-CoV-2,[17] or online adverts or surveys to assess mental health symptoms during the pandemic, excludes those without an internet connection or who do not use social media, such as older people.[18,19]

Selection bias related to who takes part in the study: Response bias occurs when people who have been invited, but do not take part in a study differ systematically from those who take part in ways that are associated with the condition of interest.[1] In the REACT-1 study,[20] for example, across four survey rounds, the investigators invited 2.4 million people; 596,000 swabs that were returned had a valid result (25%). Response rates were lower in later than in earlier rounds, in men than women and in younger than older age groups. The observed results could be weighted statistically to represent the overall characteristics of the target population but might still be biased by unmeasurable characteristics that drive willingness to take part.

The direction of response bias is often not predictable (can result in over-or underestimation of the true prevalence) because information about the motivation to take part in a study, or not, is not usually collected.[13] In a multicentre cross-sectional survey of the prevalence of PCR-determined SARS-CoV-2 in hospitals in England, the authors suggested that different selection biases could have had opposing effects.[21] For example, staff might have volunteered to take part if they were concerned that they might have been exposed to COVID-19. If such people were more likely than unexposed people to be tested,

For peer review only

prevalence might be over-estimated. Alternatively, workers in lower-paid jobs, without financial support might have been less likely to take part than those at higher grades because of the consequences for themselves or their contacts if found to be infected. If the less-well paid jobs are also associated with a higher risk of exposure to SARS-CoV-2, the prevalence in the study population would be underestimated. Accorsi et al. suggest that the risk of non-response bias in seroprevalence studies might be reduced by sampling from established and well-characterised cohorts with high rates of participation, in whom the characteristics of non-respondents are known.[13]

As the proportion of invited people that do not take part in a study increases, the probability of response bias might also increase if the topic of the study influences the probability of participation and the composition of the study population.[22] Empirical evidence of bias was found in a systematic review of sexually transmitted *Chlamydia trachomatis* infection; prevalence surveys with the lowest response rates found the highest prevalence of infection, suggesting selective participation by those with a high risk of being infected.[23] Whether or not there is a dose-response relationship between survey response rate and the likelihood of SARS-CoV-2 infection is unclear. The risks of selection bias at the stages of invitation and participation can be interrelated and might oppose each other. In the REACT-1 study,[20] it is not clear whether the reduction in selection bias through random sampling outweighed the potential for selection bias owing to the high and increasing response rate over time, or vice versa.

For peer review only

Information bias

Information bias occurs when there are systematic errors affecting the completeness or accuracy of the measurement of the condition or risk factor of interest.[1] There are different types of information bias.

Misclassification bias: This refers to the incorrect classification of a participant as having, or not having, the condition of interest.[1] Misclassification is an important source of measurement bias in prevalence studies because diagnostic tests are imperfect and might not distinguish clearly among those with and without the condition.[24] Seroprevalence studies are essential for determining the proportion of a population that has been exposed to SARS-CoV-2 up to a given time point. Detection of antibodies is affected by the test type and manufacturer, sample type such as serum, dried blood

For peer review only - http://bmjopen.bmj.com/site/about/guidelines.xhtml

spots, saliva, urine or others,[25 26] and the time of sampling after infection. Accorsi et al. have described in detail the biases in the ascertainment of SARS-CoV-2 seroprevalence studies.[13] For diagnostic tests, the predictive values will be influenced by the prevalence of the condition in the study population. Test accuracy can also change across populations, owing to the inherent characteristics of tests when clinical variability is present,[27] for example when tests for SARS-CoV-2 detection are applied to people with or without symptoms.

In a new disease, such as COVID-19, diagnostic criteria might not be standardised or might change over time. For example, accurate assessment of the prevalence of persistent asymptomatic SARS-CoV-2 infection requires a complete list of symptoms and follow-up for a sufficiently long duration to ensure that symptoms did not develop later.[15 28] In a prevalence study conducted in a nursing facility in March 2020, patients were asked about typical and non-typical symptoms of COVID-19. However, symptoms such as anosmia or ageusia had not been reported in association with SARS-CoV-2 at that time, so patients with these as isolated symptoms could have been wrongly classified as asymptomatic.[15 29] Poor quality of data collection has also been found in studies estimating the prevalence of mental health problems during the pandemic.[30] The use of non-validated scales, or dichotomisation to define the cases using inappropriate or unclear thresholds, will bias the estimated prevalence of the condition. Misclassification may also occur in calculations of the secondary attack rate (the prevalence of SARS-CoV-2 in contacts of diagnosed cases) if not all contacts are tested, and it is assumed that individuals that were not tested were also uninfected.[13]

Recall bias: this bias results in misclassification when the condition has been measured through surveys or questionnaires that rely on memory.[1] A study that aimed to describe the characteristics and symptom profile of individuals with SARS-CoV-2 infection in the United States collected information about symptoms before, and for 14 days after, being enrolled in the study.[31] The authors discuss the potential for recall bias when collecting symptoms retrospectively and if different people recollect different symptoms.

Observer bias: This bias occurs when an observer provides a wrong measurement due to lack of training or subjectivity.[1] For example, a study in the United States of America found variation between

For peer review only - http://bmjopen.bmj.com/site/about/guidelines.xhtml

14 universities in the prevalence of clinical and subclinical myocarditis in competitive athletes with SARS-CoV-2 infection.[32] One of the diagnostic tools was cardiac magnetic resonance and authors attributed some of the observer variation to differences in the protocols and the expertise among assessors. To reduce the risk of observer bias, researchers should aim to use tools that minimise subjectivity and standardise training procedures.

Reporting studies of prevalence

There is no agreed list of preferred items for reporting studies of prevalence. The published article or a preprint are usually the only available record of a study to which most people, other than the investigators themselves, have access. This written report therefore needs to contain the information required to understand the possible biases and assess internal and external validity. The Strengthening the Reporting of Observational Studies in Epidemiology (STROBE) statement is a widely used guideline, which includes recommendations for cross-sectional studies that examine associations between an exposure and outcome.[33] We refer to selected STROBE statement items and recommendations for cross-sectional studies, where they are relevant to the complete and transparent description of studies of prevalence.

First, clear definitions of the target population, study setting and eligibility criteria to select the study population are required (STROBE items 5, 6a). These issues affect assessment of external validity[34] because estimates of prevalence in a specific population and setting are often generalised more widely.[1 14] Second, the denominator used to calculate the prevalence should be clearly stated, with a description of each stage of the study showing the numbers of individuals eligible, included, and analysed (STROBE item 13a). Accurate reports of the numbers and characteristics of those who take part (responders) or do not take part (non-responders) in the study are needed for the assessment of selection bias.[22 35] Poor reporting about response rate has been described as one of the main limitations of studies in systematic reviews of prevalence.[36] As with reports of studies of any design, the statistical methods applied to provide prevalence estimates (STROBE item 12d) need to be reported clearly.[33] The setting, location and periods of enrolment and data collection (STROBE item 5) are particularly important for studies of SARS-CoV-2; the stage of the pandemic, preventive measures in place and

For peer review only - http://bmjopen.bmj.com/site/about/guidelines.xhtml

virus variants in circulation should all be described because these affect the interpretation of estimates of prevalence. Third, it is crucial to provide a clear definition of the condition or risk factor of interest (STROBE item 7) and how it was measured, so that the risk of information bias can be assessed. The definition may be straightforward if there are objective criteria for ascertainment. For example, studies of the prevalence of active SARS-CoV-2 infection should report the diagnostic test, manufacturer, sample type, and criteria for a positive result.[37 38] For new conditions that have not been fully characterised, such as post COVID-19 condition, also known as 'long COVID', reporting of prevalence can be challenging.[39 40] The World Health Organization produced a case definition[41] in October 2021, but this might take time to be adopted widely.

Conclusion

The experience of the COVID-19 pandemic has highlighted the importance of biases that affect the measurement of prevalence and reporting quality. Biased results from prevalence studies can have a direct impact at the individual and community level. In studies of prevalence, biases affecting internal validity can also affect the external validity of findings when estimates from a study population are interpreted as being representative and generalised to the target population.[34]

It is vital to increase research efforts on bias components, study designs, and reporting of prevalence studies. Some questions about prevalence need to be addressed through systematic reviews and meta-epidemiological studies. A high proportion of published systematic reviews of prevalence, however, also have flaws in reporting and methodological quality.[5 42] Confidence in the results of systematic reviews is determined by the credibility of the primary studies and the methods used to synthesise them. The concepts and examples provided here can be applied to any condition or outcome. The volume of research published during the COVID-19 pandemic has put the spotlight on research methods, interpretation of the risks of bias and reporting of studies of prevalence. Efforts to strengthen methodological research about, and improve the quality of, studies of prevalence in all fields of research should continue beyond this pandemic.

For peer review only - http://bmjopen.bmj.com/site/about/guidelines.xhtml

Figure legend

Potential for selection and information bias in prevalence studies. Coloured lines relate to the coloured boxes, showing at which stage of study procedures selection bias (blue line) and information bias (purple line) can occur.

References

1. Porta M. A Dictionary of Epidemiology Oxford, United States: Oxford University Press; 2014 [6th Edition:[Available from: https://www.oxfordreference.com/view/10.1093/acref/9780195314496.001.0001/acref-9780195314496. (Accessed 24 March 2022).

2. Rothman KJ, Greenland S, Lash TL. Measures of Occurrence In: Rothman KJ, Greenland S, eds. Modern Epidemiology. Boston, Massachusetts: Lippincott Williams & Wilkins 2020:32-50.

3. Institute for Health Metrics and Evaluation (IHME). The Global Burden of Disease Study: University of Washington; 2020 Available from: http://www.healthdata.org/gbd/about (Accessed 24 March 2022).

4. Vos T, Lim SS, Abbafati C, et al. Global burden of 369 diseases and injuries in 204 countries and territories, 1990-2019: a systematic analysis for the Global Burden of Disease Study 2019. *Lancet* 2020;396(10258):1204-22.

5. Hoffmann F, Eggers D, Pieper D, et al. An observational study found large methodological heterogeneity in systematic reviews addressing prevalence and cumulative incidence. *J Clin Epidemiol* 2020;119:92-99. https://www.sciencedirect.com/science/article/pii/S0895435619308820 (Accessed 5 April 2022).

6. Egger M, Davey-Smith G, Altman D. Systematic Reviews in Health Care: Meta-Analysis in Context. 2nd ed: Wiley 2001.

7. Higgins JPT, Thomas J, Chandler J, et al. Cochrane Handbook for Systematic Reviews of Interventions Version 6.2 (updated February 2021): Cochrane; 2021 [Available from: www.training.cochrane.org/handbook. (Accessed 24 March 2022).

8. Lipsitch M, Swerdlow DL, Finelli L. Defining the Epidemiology of Covid-19 — Studies Needed. *N Engl J Med* 2020;382(13):1194-96.

9. Riley S, Atchison C, Ashby D, et al. REal-time Assessment of Community Transmission (REACT) of SARS-CoV-2 virus: Study protocol. *Wellcome Open Res* 2021;5:200. https://www.ncbi.nlm.nih.gov/pmc/articles/PMC8095190/ (Accessed 5 April 2022).

10. World Health Organization. Population-based age-stratified seroepidemiological investigation protocol for coronavirus 2019 (COVID-19) infection. Version 2.0. Geneva, 2020.

11. Siegler AJ, Sullivan PS, Sanchez T, et al. Protocol for a national probability survey using home specimen collection methods to assess prevalence and incidence of SARS-CoV-2 infection and antibody response. *Ann Epidemiol* 2020;49:50-60. https://www.sciencedirect.com/science/article/pii/S1047279720302751 (Accessed 5 April 2022).

For peer review only - http://bmjopen.bmj.com/site/about/guidelines.xhtml

12. Levin AT, Hanage WP, Owusu-Boaitey N, et al. Assessing the age specificity of infection fatality rates for COVID-19: systematic review, meta-analysis, and public policy implications. *Eur J Epidemiol* 2020;35(12):1123-38. https://www.ncbi.nlm.nih.gov/pubmed/33289900 (Accessed 5 April 2022).

13. Accorsi EK, Qiu X, Rumpler E, et al. How to detect and reduce potential sources of biases in studies of SARS-CoV-2 and COVID-19. *Eur J Epidemiol* 2021:179-96. (Accessed 25 Feb 2022).

14. Griffith GJ, Morris TT, Tudball MJ, et al. Collider bias undermines our understanding of COVID-19 disease risk and severity. *Nat Commun* 2020;11(1):5749. https://doi.org/10.1038/s41467-020-19478-2 (Accessed 5 April 2022).

15. Meyerowitz EA, Richterman A, Bogoch I, et al. Towards an accurate and systematic characterisation of persistently asymptomatic infection with SARS-CoV-2. *Lancet Infect Dis* 2021;21(6):e163-e69.

16. Simera I, Moher D, Hirst A, et al. Transparent and accurate reporting increases reliability, utility, and impact of your research: reporting guidelines and the EQUATOR Network. *BMC Med* 2010;8:24. https://pubmed.ncbi.nlm.nih.gov/20420659 (Accessed 5 April 2022).

17. Bendavid E, Mulaney B, Sood N, et al. COVID-19 antibody seroprevalence in Santa Clara County, California. *Int J Epidemiol* 2021;50(2):410-19.

18. Abir T, Kalimullah NA, Osuagwu UL, et al. Prevalence and Factors Associated with Mental Health Impact of COVID-19 Pandemic in Bangladesh: A Survey-Based Cross-Sectional Study. *Ann Glob Health* 2021;87(1):43. https://pubmed.ncbi.nlm.nih.gov/33981590 (Accessed 4 April 2022).

19. De Man J, Campbell L, Tabana H, et al. The pandemic of online research in times of COVID-19. *BMJ Open* 2021;11(2):e043866. http://bmjopen.bmj.com/content/11/2/e043866.abstract (Accessed 5 April 2022).

20. Riley S, Wang H, Eales O, et al. REACT-1 round 12 report: resurgence of SARS-CoV-2 infections in England associated with increased frequency of the Delta variant. *medRxiv* 2021:2021.06.17.21259103. http://medrxiv.org/content/early/2021/06/21/2021.06.17.21259103.abstract (Accessed 5 April 2022).

21. Brown CS, Clare K, Chand M, et al. Snapshot PCR surveillance for SARS-CoV-2 in hospital staff in England. *J Infect* 2020;81(3):427-34. https://www.sciencedirect.com/science/article/pii/S0163445320304503 (Accessed 5 April 2022).

22. Groves RM, Peytcheva E. The Impact of Nonresponse Rates on Nonresponse Bias: A Meta-Analysis. *Public Opin Q* 2008;72(2):167-89. http://www.jstor.org/stable/25167621 (Accessed 5 April 2022).

23. Redmond SM, Alexander-Kisslig K, Woodhall SC, et al. Genital Chlamydia prevalence in europe and non-European high income countries: systematic review and meta-analysis. *PLoS One* 2015;10(1):e0115753. http://www.ncbi.nlm.nih.gov/pubmed/25615574 (Accessed 5 April 2022).

24. Lord SJ, Staub LP, Bossuyt PMM, et al. Target practice: choosing target conditions for test accuracy studies that are relevant to clinical practice. *BMJ* 2011;343:d4684. https://www.bmj.com/content/bmj/343/bmj.d4684.full.pdf (Accessed 5 April 2022).

For peer review only

25. Jeong HW, Kim S-M, Kim H-S, et al. Viable SARS-CoV-2 in various specimens from COVID-19 patients. *Clin Microbiol Infect* 2020;26(11):1520-24. https://www.sciencedirect.com/science/article/pii/S1198743X20304274 (Accessed 5 April 2022).

26. Niedrig M, Patel P, El Wahed AA, et al. Find the right sample: A study on the versatility of saliva and urine samples for the diagnosis of emerging viruses. *BMC Infect Dis* 2018;18(1):707. https://doi.org/10.1186/s12879-018-3611-x (Accessed 5 April 2022).

27. Leeflang MMG, Bossuyt PMM, Irwig L. Diagnostic test accuracy may vary with prevalence: implications for evidence-based diagnosis. *J Clin Epidemiol* 2009;62(1):5-12. https://doi.org/10.1016/j.jclinepi.2008.04.007 (Accessed 5 April 2022).

28. Buitrago-Garcia D, Egli-Gany D, Counotte MJ, et al. Occurrence and transmission potential of asymptomatic and presymptomatic SARS-CoV-2 infections: A living systematic review and meta-analysis. *PLoS Med* 2020;17(9):e1003346. https://doi.org/10.1371/journal.pmed.1003346 (Accessed 5 April 2022).

29. Kimball A, Hatfield K, Arons M, et al. Asymptomatic and Presymptomatic SARS-CoV-2 Infections in Residents of a Long-Term Care Skilled Nursing Facility — King County, Washington, March 2020. *MMWR Morb Mortal Wkly Rep* 2020;69.

30. Lin YH, Chen CY, Wu SI. Efficiency and Quality of Data Collection Among Public Mental Health Surveys Conducted During the COVID-19 Pandemic: Systematic Review. *J Med Internet Res* 2021;23(2):e25118.

31. Yousaf AR, Duca LM, Chu V, et al. A prospective cohort study in non-hospitalized household contacts with SARS-CoV-2 infection: symptom profiles and symptom change over time. *Clin Infect Dis* 2020. https://www.ncbi.nlm.nih.gov/pubmed/32719874 (Accessed 5 April 2022).

32. Daniels CJ, Rajpal S, Greenshields JT, et al. Prevalence of Clinical and Subclinical Myocarditis in Competitive Athletes With Recent SARS-CoV-2 Infection: Results From the Big Ten COVID-19 Cardiac Registry. *JAMA Cardiol* 2021;6(9):1078-1087.

33. Vandenbroucke JP, von Elm E, Altman DG, et al. Strengthening the Reporting of Observational Studies in Epidemiology (STROBE): explanation and elaboration. *Int J Surg* 2014;12(12):1500-24.

34. Galea S, Tracy M. Participation rates in epidemiologic studies. *Ann Epidemiol* 2007;17(9):643-53. https://www.ncbi.nlm.nih.gov/pubmed/17553702 (Accessed 5 April 2022).

35. Groves RM. Nonresponse Rates and Nonresponse Bias in Household Surveys. *Public Opin Q* 2006;70(5):646-75. http://www.jstor.org/stable/4124220 (Accessed 5 April 2022).

36. Baumann L, Cina M, Egli-Gany D, et al. Prevalence of Mycoplasma genitalium in different population groups: systematic review andmeta-analysis. *Sex Transm Infect* 2018;94(4):255-62.

37. Chu DKW, Pan Y, Cheng SMS, et al. Molecular Diagnosis of a Novel Coronavirus (2019-nCoV) Causing an Outbreak of Pneumonia. *Clin Chem* 2020;66(4):549-55.

38. Corman VM, Landt O, Kaiser M, et al. Detection of 2019 novel coronavirus (2019-nCoV) by real-time RT-PCR. *Euro Surveill* 2020;25(3):2000045. https://www.eurosurveillance.org/content/10.2807/1560-7917.ES.2020.25.3.2000045 Accessed 5 April 2022).

39. Whitaker M, Elliott J, Chadeau-Hyam M, et al. Persistent symptoms following SARS-CoV-2 infection in a random community sample of 508,707 people. *medRxiv*

For peer review only

2021:2021.06.28.21259452. http://medrxiv.org/content/early/2021/07/03/2021.06.28.21259452.abstract (Accessed 5 April 2022).

40. Michelen M, Manoharan L, Elkheir N, et al. Characterising long COVID: a living systematic review. *BMJ Glob Health* 2021;6(9):e005427. https://gh.bmj.com/content/6/9/e005427 (Accessed 5 April 2022).

41. World Health Organization. A clinical case definition of post COVID-19 condition by a Delphi consensus 2021 [updated 6 October 2021. Available from: https://www.who.int/publications/i/item/WHO-2019-nCoV-Post_COVID-19_condition-Clinical_case_definition-2021.1 (Accessed 5 April 2022).

42. Page MJ, McKenzie JE, Kirkham J, et al. Bias due to selective inclusion and reporting of outcomes and analyses in systematic reviews of randomised trials of healthcare interventions. *Cochrane Database Syst Rev* 2014(10):Mr000035.

Declarations

Acknowledgment: We thank Yuli Baron Castillo for producing Figure 1.

Funding: This work was supported by the Swiss government excellence scholarship Grant number [2019.0774], the SSPH+ Global PhD Fellowship Programme in Public Health Sciences (GlobalP3HS) of the Swiss School of Public Health, and the Swiss National Science Foundation (project grant 176233).

Authors' contributions: Diana Buitrago-Garcia, Georgia Salanti and Nicola Low conceptualised the project. Diana Buitrago-Garcia wrote the first draft of the manuscript. Georgia Salanti and Nicola Low provided input to revise the manuscript. Georgia Salanti and Nicola Low supervised the research. All approved the final manuscript.

Ethics approval: Not applicable

Consent to participate: Not applicable

Consent for publication: Not applicable

For peer review only

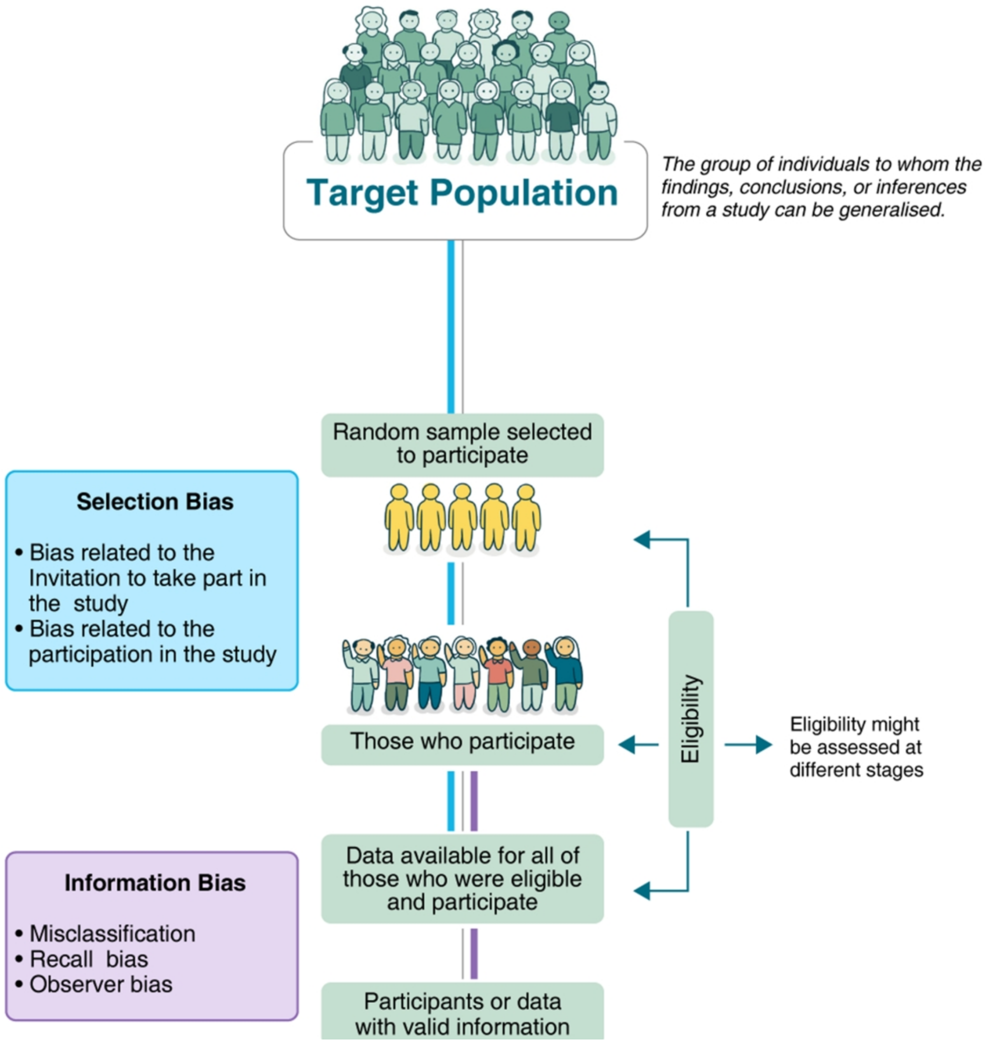

Potential for selection and information bias in prevalence studies. Coloured lines relate to the coloured boxes, showing at which stage of study procedures selection bias (blue line) and information bias (purple line) can occur.

433x458mm (118 x 118 DPI)

BMJ Open

Studies of prevalence. How a basic epidemiology concept has gained recognition in the COVID-19 pandemic.

Journal:	*BMJ Open*
Manuscript ID	bmjopen-2022-061497.R2
Article Type:	Communication
Date Submitted by the Author:	23-Aug-2022
Complete List of Authors:	Buitrago-Garcia, Diana; University of Bern Faculty of Medicine, Institute of Social and Preventive Medicine (ISPM); University of Bern, Graduate School of Health Sciences<br>Salanti, Georgia; University of Bern Faculty of Medicine, Institute of Social and Preventive Medicine<br>Low, Nicola; University of Bern Faculty of Medicine, Institute of Social and Preventive Medicine
**Primary Subject Heading**:	Epidemiology
Secondary Subject Heading:	Infectious diseases
Keywords:	EPIDEMIOLOGY, COVID-19, STATISTICS & RESEARCH METHODS

SCHOLARONE™ Manuscripts

I, the Submitting Author has the right to grant and does grant on behalf of all authors of the Work (as defined in the below author licence), an exclusive licence and/or a non-exclusive licence for contributions from authors who are: i) UK Crown employees; ii) where BMJ has agreed a CC-BY licence shall apply, and/or iii) in accordance with the terms applicable for US Federal Government officers or employees acting as part of their official duties; on a worldwide, perpetual, irrevocable, royalty-free basis to BMJ Publishing Group Ltd ("BMJ") its licensees and where the relevant Journal is co-owned by BMJ to the co-owners of the Journal, to publish the Work in BMJ Open and any other BMJ products and to exploit all rights, as set out in our licence.

The Submitting Author accepts and understands that any supply made under these terms is made by BMJ to the Submitting Author unless you are acting as an employee on behalf of your employer or a postgraduate student of an affiliated institution which is paying any applicable article publishing charge ("APC") for Open Access articles. Where the Submitting Author wishes to make the Work available on an Open Access basis (and intends to pay the relevant APC), the terms of reuse of such Open Access shall be governed by a Creative Commons licence – details of these licences and which Creative Commons licence will apply to this Work are set out in our licence referred to above.

Other than as permitted in any relevant BMJ Author's Self Archiving Policies, I confirm this Work has not been accepted for publication elsewhere, is not being considered for publication elsewhere and does not duplicate material already published. I confirm all authors consent to publication of this Work and authorise the granting of this licence.

For peer review only

Studies of prevalence. How a basic epidemiology concept has gained recognition in the COVID-19 pandemic

Diana Buitrago-Garcia

Institute of Social and Preventive Medicine, Graduate School of Health Sciences, University of Bern, Bern, Switzerland (0000-0001-9761-206X)

Email: diana.buitragogarcia@ispm.unibe.ch

Georgia Salanti

Institute of Social and Preventive Medicine, University of Bern, Bern, Switzerland (0000-0002-3830-8508)

Email: georgia.salanti@ispm.unibe.ch

Nicola Low

Institute of Social and Preventive Medicine, University of Bern, Bern, Switzerland (0000-0003-4817-8986)

Email: nicola.low@ispm.unibe.ch

Keywords

Prevalence, Observational Study, Bias, Selection Bias, Epidemiology, COVID-19

Main text 2849 words, 1 figure, 1 table

For peer review only

For peer review only - http://bmjopen.bmj.com/site/about/guidelines.xhtml

Abstract

Prevalence measures the occurrence of any health condition, exposure, or other factors related to health. The experience of COVID-19, a new disease caused by severe acute respiratory syndrome coronavirus 2 (SARS-CoV-2), has highlighted the importance of prevalence studies, for which issues of reporting and methodology have traditionally been neglected. This commentary highlights key issues about risks of bias in the design and conduct of prevalence studies and in reporting them, using examples about SARS-CoV-2 and COVID-19. The two main domains of bias in prevalence studies are those related to the study population (selection bias) and the condition or risk factor being assessed (information biases). Sources of selection bias should be considered both at the time of the invitation to take part in a study and when assessing who participates and provides valid data (respondents and non-respondents). Information bias appears when there are systematic errors affecting the accuracy and reproducibility of the measurement of the condition or risk factor. Types of information bias include misclassification, observer and recall bias. When reporting prevalence studies, clear descriptions of the target population, study population, study setting and context, and clear definitions of the condition or risk factor and its measurement, are essential. Without clear reporting, the risks of bias cannot be assessed properly. Bias in the findings of prevalence studies can, however, impact decision-making and the spread of disease. The concepts discussed here can be applied to the assessment of prevalence for many other conditions. Efforts to strengthen methodological research about, and improve the quality of reporting of, studies of prevalence in all fields of research should continue beyond this pandemic.

For peer review only - http://bmjopen.bmj.com/site/about/guidelines.xhtml

In introductory epidemiology, students learn about prevalence, an easy to understand concept, defined as "*a proportion that measures disease occurrence of any type of health condition, exposure, or other factor related to health*",[1] or "*the proportion of persons in a population who have a particular disease or attribute at a specified point in time or over a specified period.*"[2] Prevalence is an important measure for assessing the magnitude of health-related conditions, and studies of prevalence are an important source of information for estimating the burden of disease, injuries and risk factors.[3] Accurate information about prevalence enables health authorities to assess the health needs of a population, to develop prevention programmes and prioritise resources to improve public health.[4] Perhaps, owing to the apparent simplicity of the concept of prevalence, methodological developments to assess the quality of reporting, the potential for bias, and the synthesis of prevalence estimates in meta-analysis have been neglected,[5] when compared with the attention paid to methods relevant to evidence from randomised controlled trials and comparative observational studies.[6 7]

The COVID-19 pandemic has shown the need for epidemiological studies to describe and understand a new disease quickly but accurately.[8] Studies reporting on prevalence have been an important source of evidence to describe the prevalence of active SARS-CoV-2 infection and antibodies to SARS-CoV-2, the spectrum of SARS-CoV-2-related morbidity and helped to understand factors related to infection and disease to inform national decisions about containment measures.[9-11] Accurate estimates of prevalence of SARS-CoV-2 are crucial because they are used as an input for the estimation of other quantities, such as infection fatality ratios, which can be calculated indirectly using seroprevalence estimates.[12] Assessments of published studies have, however, highlighted methodological issues that affect study design, conduct, analysis, interpretation, and reporting.[13-15] In addition, some questions about prevalence need to be addressed through systematic reviews and meta-epidemiological studies. A high proportion of published systematic reviews of prevalence, however, also have flaws in reporting and methodological quality.[5 16] Confidence in the results of systematic reviews is determined by the credibility of the primary studies and the methods used to synthesise them.

The objective of this communication is to highlight key issues about the risk of bias in studies that measure prevalence and about the quality of reporting, using examples about SARS-CoV-2 and

For peer review only - http://bmjopen.bmj.com/site/about/guidelines.xhtml

COVID-19. We refer to prevalence at the level of a population, and not as a prediction at an individual level. The estimand is therefore “what proportion of the population is positive” and not “what is the probability this person is positive.” Although incidence and prevalence are related epidemiologically, we do not discuss incidence in this article because the study designs for measurement of the quantities differ. Bias is a systematic deviation of results or inferences from the underlying (unobserved) true values.[1] The risk of bias is a judgment about the degree to which the methods or findings of a study might under-or over-estimate the true value in the target population,[7] in this case, the prevalence of a condition or risk factor. Quality of reporting refers to the completeness and transparency of the presentation of a research publication.[17] Risk of bias and quality of reporting are separate, but closely related, because it is only possible to assess the strengths and weaknesses of a study report if the methods and results are described adequately.

Bias in prevalence studies

The two main domains of bias in prevalence studies are those related to the study population (selection bias) and the condition being assessed (information biases) (Fig. 1). Biases involved in the design, conduct and analysis of a study affect its internal validity. Selection bias also affects external validity, the extent to which findings from a specific study can be generalised to a wider, target population in time and space. There are many names given to different biases, often addressing the same concept. For this communication, we use the names and definitions published in the Dictionary of Epidemiology.[1]

Selection bias

Selection bias relates to the representativeness of the sample used to estimate the prevalence in relation to the target population. The target population is the group of individuals to whom the findings, conclusions, or inferences from a study can be generalised.[1] There are two steps in a prevalence study at which selection bias might occur: at the invitation to take part in the study and, among those invited, who take part (Fig. 1).

[Fig 1.]

For peer review only - http://bmjopen.bmj.com/site/about/guidelines.xhtml

Selection bias in the invitation to take part in the study: The probability of being invited to take part in a study should be the same for every person in the target population. Evaluation of selection bias at this stage should therefore account for the complexity of the strategy for identification of participants. For example, if participants are invited from people who have previously agreed to participate in a registry or cohort, each level of invitation that has contributed to the final setting should be judged for the increasing risk of self-selection. Those who are invited to take part might be defined by demographic characteristics, e.g., children below ten years or study setting (e.g., hospitalised patients), or a random sample of the general population. The least biased method to select participants in a prevalence study is to sample at random from the target population. For example, the Real-time Assessment of Community Transmission (REACT) Studies to assess the prevalence of the virus, using molecular diagnostic tests (REACT-1) and antibodies (REACT-2), invite random samples of people, stratified by area, from the National Health Service patient list in England.[9] Those invited are close to a truly random sample because almost everyone in England is registered with a general practitioner. In some cases, criteria applied to the selection of a random sample might still result in bias. For example, a seroprevalence study conducted in Spain did not include care home addresses, which could have excluded around 6% of the Spanish older population.[18] Excluding people in long term facilities might underestimate SARS-CoV-2 seroprevalence in older adults, who have been at higher risk of exposure than the average in the general population.[13] Other methods of sampling are at risk of selection bias. For example, asking for volunteers through advertisements are liable to selection bias because not everyone has the same probability of seeing or replying to the advert. For example, the use of social media to invite people to a drive-in test centre to estimate the population prevalence of antibodies to SARS-CoV-2,[19] or online adverts to assess mental health symptoms during the pandemic, excludes those without an internet connection or who do not use social media, such as older people.[20]

Selection bias related to who takes part in the study: Non-response bias occurs when people who have been invited, but do not take part in a study differ systematically from those who take part in ways that are associated with the condition of interest.[21] In the REACT-1 study,[22] for example, across four survey rounds, the investigators invited 2.4 million people; 596,000 swabs that were returned had a valid result (25%). The proportion of participants responding was lower in later than in earlier rounds,

For peer review only - http://bmjopen.bmj.com/site/about/guidelines.xhtml

in men than women and in younger than older age groups. If the sociodemographic characteristics of the target population are known, the observed results could be weighted statistically to represent the overall population but might still be biased by unmeasurable characteristics that drive willingness to take part.

The direction of non-response bias is often not predictable (can result in over-or underestimation of the true prevalence) because information about the motivation to take part in a study, or not, is not usually collected.[13] In a multicentre cross-sectional survey of the prevalence of PCR-determined SARS-CoV-2 in hospitals in England, the authors suggested that different selection biases could have had opposing effects.[23] For example, staff might have volunteered to take part if they were concerned that they might have been exposed to COVID-19. If such people were more likely than unexposed people to be tested, prevalence might be over-estimated. Alternatively, workers in lower-paid jobs, without financial support might have been less likely to take part than those at higher grades because of the consequences for themselves or their contacts if found to be infected. If the less-well paid jobs are also associated with a higher risk of exposure to SARS-CoV-2, the prevalence in the study population would be underestimated. Accorsi et al. suggest that the risk of non-response bias in seroprevalence studies might be reduced by sampling from established and well-characterised cohorts with high rates of participation, in whom the characteristics of non-respondents are known.[13]

As the proportion of invited people that do not take part in a study (the non-response rate) increases, the probability of non-response bias might also increase if the topic of the study influences the probability and the composition of the study populationy.[24] Empirical evidence of bias was found in a systematic review of sexually transmitted *Chlamydia trachomatis* infection; prevalence surveys with the lowest response rates found the highest prevalence of infection, suggesting selective participation by those with a high risk of being infected.[25] Whether or not there is a dose-response relationship between survey response rate and the likelihood of SARS-CoV-2 infection is unclear. The risks of selection bias at the stages of invitation and participation can be interrelated and might oppose each other. In the REACT-1 study,[22] it is not clear whether the reduction in selection bias through random

For peer review only - http://bmjopen.bmj.com/site/about/guidelines.xhtml

sampling outweighed the potential for selection bias owing to the high and increasing non-response rate over time, or vice versa.

Information bias

Information bias occurs when there are systematic errors affecting the completeness or accuracy of the measurement of the condition or risk factor of interest. There are different types of information bias.

Misclassification bias: This refers to the incorrect classification of a participant as having, or not having, the condition of interest. Misclassification is an important source of measurement bias in prevalence studies because diagnostic tests are imperfect and might not distinguish clearly among those with and without the condition.[26] For diagnostic tests, the predictive values will also be influenced by the prevalence of the condition in the study population. Seroprevalence studies are essential for determining the proportion of a population that has been exposed to SARS-CoV-2 up to a given time point. Detection of antibodies is affected by the test type and manufacturer, sample type such as serum, dried blood spots, saliva, urine or others,[27 28] and the time of sampling after infection. Different diagnostic tests might also be used in participants in the same study population, but adjustment for test performance is not always appropriate because the characteristics derived from studies in which the tests were validated might differ from the study population.[13] Accorsi et al. have described in detail this issue and other biases in the ascertainment of SARS-CoV-2 seroprevalence studies.[13] Test accuracy can also change across populations, owing to the inherent characteristics of tests when clinical variability is present,[29] for example when tests for SARS-CoV-2 detection are applied to people with or without symptoms.

In a new disease, such as COVID-19, diagnostic criteria might not be standardised or might change over time. For example, accurate assessment of the prevalence of persistent asymptomatic SARS-CoV-2 infection requires a complete list of symptoms and follow-up for a sufficiently long duration to ensure that symptoms did not develop later.[15 30] In a prevalence study conducted in a nursing facility in March 2020, patients were asked about typical and non-typical symptoms of COVID-19. However, symptoms such as anosmia or ageusia had not been reported in association with SARS-CoV-2 at that time, so patients with these as isolated symptoms could have been wrongly classified as

For peer review only - http://bmjopen.bmj.com/site/about/guidelines.xhtml

asymptomatic.[15 31] Poor quality of data collection has also been found in studies estimating the prevalence of mental health problems during the pandemic.[32] The use of non-validated scales, or dichotomisation to define the cases using inappropriate or unclear thresholds, will bias the estimated prevalence of the condition. Misclassification may also occur in calculations of the secondary attack rate (the prevalence of SARS-CoV-2 in contacts of diagnosed cases) if not all contacts are tested, and it is assumed that individuals that were not tested were also uninfected.[13]

Recall bias: this bias results in misclassification when the condition has been measured through surveys or questionnaires that rely on memory. A study that aimed to describe the characteristics and symptom profile of individuals with SARS-CoV-2 infection in the United States collected information about symptoms before, and for 14 days after, being enrolled in the study.[33] The authors discuss the potential for recall bias when collecting symptoms retrospectively and if different people recollect different symptoms.

Observer bias: This bias occurs when an observer provides a wrong measurement due to lack of training or subjectivity.[21] For example, a study in the United States of America found variation between 14 universities in the prevalence of clinical and subclinical myocarditis in competitive athletes with SARS-CoV-2 infection.[34] One of the diagnostic tools was cardiac magnetic resonance and authors attributed some of the variability to differences in the protocols and the expertise among assessors. To reduce the risk of observer bias, researchers should aim to use tools that minimise subjectivity and standardise training procedures.

Reporting studies of prevalence

There is no agreed list of preferred items for reporting studies of prevalence. The published article or a preprint are usually the only available record of a study to which most people, other than the investigators themselves, have access. The written report therefore needs to contain the information required to understand the possible biases and assess internal and external validity. The Strengthening the Reporting of Observational Studies in Epidemiology (STROBE) statement is a widely used guideline, which includes recommendations for cross-sectional studies that examine associations between an exposure and outcome.[35] Table 1 shows selected items from the STROBE statement and

For peer review only - http://bmjopen.bmj.com/site/about/guidelines.xhtml

recommendations for cross-sectional studies that are particularly relevant to the complete and transparent description of methods for studies of prevalence.

Table 1. Items from the STROBE checklist for cross-sectional studies that are relevant for prevalence studies

Item	Item number	Recommendation
Setting	5	Describe the setting, locations, and relevant dates, including periods of recruitment, exposure, follow-up, and data collection
Participants	6a	Give the eligibility criteria, and the sources and methods of selection of participants
Variables	7	Clearly define all outcomes, exposures, predictors, potential confounders, and effect modifiers. Give diagnostic criteria, if applicable
Data sources/ measurement	8	For each variable of interest, give sources of data and details of methods of assessment (measurement). Describe comparability of assessment methods if there is more than one group.
Statistical methods	12c	Explain how missing data were addressed
	12d	If applicable, describe analytical methods taking account of sampling strategy
Participants	13a	Report numbers of individuals at each stage of study—eg numbers potentially eligible, examined for eligibility, confirmed eligible, included in the study, completing follow-up, and analysed
	13b	Give reasons for non-participation at each stage

First, clear definitions of the target population, study setting and eligibility criteria to select the study population are required (STROBE items 5, 6a). These issues affect assessment of external validity[36] because estimates of prevalence in a specific population and setting are often generalised more widely.[1 14] Second, the denominator used to calculate the prevalence should be clearly stated, with a

For peer review only - http://bmjopen.bmj.com/site/about/guidelines.xhtml

description of each stage of the study showing the numbers of individuals eligible, included, and analysed (STROBE item 13a, b). Accurate reports of the numbers and characteristics of those who take part (responders) or do not take part (non-responders) in the study are needed for the assessment of selection bias, but this information is not always available.[24 37] Poor reporting about the proportion of responders has been described as one of the main limitations of studies in systematic reviews of prevalence.[38] As with reports of studies of any design, the statistical methods applied to provide prevalence estimates, including methods used to address missing data (STROBE item 12c) and to account for the sampling strategy (STROBE item 12d) need to be reported clearly.[35] The setting, location and periods of enrolment and data collection (STROBE item 5) are particularly important for studies of SARS-CoV-2; the stage of the pandemic, preventive measures in place and virus variants in circulation should all be described because these affect the interpretation of estimates of prevalence. Third, it is crucial to provide a clear definition of the condition or risk factor of interest (STROBE item 7) and how it was measured (STROBE item 8), so that the risk of information bias can be assessed. The definition may be straightforward if there are objective criteria for ascertainment. For example, studies of the prevalence of active SARS-CoV-2 infection should report the diagnostic test, manufacturer, sample type, and criteria for a positive result.[39 40] For new conditions that have not been fully characterised, such as post COVID-19 condition, also known as 'long COVID', reporting of prevalence can be challenging.[41 42] The World Health Organization produced a case definition[43] in October 2021, but this might take time to be adopted widely.

Conclusion

The COVID-19 pandemic has produced an enormous amount of research about a single disease, published over a short time period.[44 45] Authors who have assessed the body of research on COVID-19 have highlighted concerns about the risks of bias in different study designs, including studies of prevalence.[13 44] In systematic reviews of a single topic, the occurrence of asymptomatic SARS-CoV-2 infection, we observed high between-study heterogeneity, serious risks of bias and poor reporting in the measurement of prevalence.[30] Biased results from prevalence studies can have a direct impact at the levels of the individual, community, global health, and policymaking. This communication

For peer review only

For peer review only - http://bmjopen.bmj.com/site/about/guidelines.xhtml

describes concepts about risks of bias and provides examples that authors can apply to the assessment of prevalence for many other conditions. Future research should be conducted to investigate sources of bias in studies of prevalence and empirical evidence of their influence on estimates of prevalence. The development of a tool that can be adapted to assess the risk of bias in studies of prevalence, and reporting guidelines for studies of prevalence would help to improve the quality of published studies of prevalence in all fields of research beyond this pandemic.

Figure legend

Potential for selection bias in prevalence studies. Coloured lines relate to the coloured boxes, showing at which stage of study procedures selection bias (blue line) and information bias (purple line) can occur.

For peer review only

For peer review only - http://bmjopen.bmj.com/site/about/guidelines.xhtml

References

1. Porta M. A Dictionary of Epidemiology Oxford, United States: Oxford University Press; 2014 [6th Edition:[Available from: https://www.oxfordreference.com/view/10.1093/acref/9780195314496.001.0001/acref-9780195314496. accessed 24 March 2022.

2. Rothman KJ, Greenland S, Lash TL. Measures of Occurrence In: Rothman KJ, Greenland S, eds. Modern Epidemiology. Boston, Massachusetts: Lippincott Williams & Wilkins 2020:32-50.

3. Institute for Health Metrics and Evaluation (IHME). The Global Burden of Disease Study: University of Washington; 2020 [Available from: http://www.healthdata.org/gbd/about accessed 24 March 2022.

4. Vos T, Lim SS, Abbafati C, et al. Global burden of 369 diseases and injuries in 204 countries and territories, 1990-2019: a systematic analysis for the Global Burden of Disease Study 2019. *Lancet* 2020;396(10258):1204-22. doi: https://doi.org/10.1016/S0140-6736(20)30925-9

5. Hoffmann F, Eggers D, Pieper D, et al. An observational study found large methodological heterogeneity in systematic reviews addressing prevalence and cumulative incidence. *J Clin Epidemiol* 2020;119:92-99. doi: https://doi.org/10.1016/j.jclinepi.2019.12.003

6. Egger M, Davey-Smith G, Altman D. Systematic Reviews in Health Care: Meta-Analysis in Context. 2nd ed: Wiley 2001.

7. Higgins JPT, Thomas J, Chandler J, et al. Cochrane Handbook for Systematic Reviews of Interventions Version 6.2 (updated February 2021): Cochrane; 2021 [Available from: www.training.cochrane.org/handbook. accessed 24 March 2022.

8. Lipsitch M, Swerdlow DL, Finelli L. Defining the Epidemiology of Covid-19 — Studies Needed. *New England Journal of Medicine* 2020;382(13):1194-96. doi: https://doi/full/10.1056/NEJMp2002125

9. Riley S, Atchison C, Ashby D, et al. REal-time Assessment of Community Transmission (REACT) of SARS-CoV-2 virus: Study protocol. *Wellcome Open Res* 2021;5:200. doi: https://doi.org/10.12688/wellcomeopenres.16228.2

10. World Health Organization. Population-based age-stratified seroepidemiological investigation protocol for coronavirus 2019 (COVID-19) infection. Version 2.0. Geneva, 2020.

11. Siegler AJ, Sullivan PS, Sanchez T, et al. Protocol for a national probability survey using home specimen collection methods to assess prevalence and incidence of SARS-CoV-2 infection and antibody response. *Annals of Epidemiology* 2020;49:50-60. doi: https://doi.org/10.1016/j.annepidem.2020.07.015

12. Levin AT, Hanage WP, Owusu-Boaitey N, et al. Assessing the age specificity of infection fatality rates for COVID-19: systematic review, meta-analysis, and public policy implications. *Eur J Epidemiol* 2020;35(12):1123-38. doi: 10.1007/s10654-020-00698-1 [published Online First: 2020/12/09]

13. Accorsi EK, Qiu X, Rumpler E, et al. How to detect and reduce potential sources of biases in studies of SARS-CoV-2 and COVID-19. *European journal of epidemiology* 2021:179-96. doi: https://doi.org/10.1007/s10654-021-00727-7 [published Online First: 2021/02/27]

For peer review only

14. Griffith GJ, Morris TT, Tudball MJ, et al. Collider bias undermines our understanding of COVID-19 disease risk and severity. *Nature Communications* 2020;11(1):5749. doi: https://doi.org/10.1038/s41467-020-19478-2

15. Meyerowitz EA, Richterman A, Bogoch I, et al. Towards an accurate and systematic characterisation of persistently asymptomatic infection with SARS-CoV-2. *The Lancet Infectious diseases* 2021;21(6):e163-e69. doi: https://doi.org/10.1016/S1473-3099(20)30837-9 [published Online First: 2020/12/11]

16. Page MJ, McKenzie JE, Kirkham J, et al. Bias due to selective inclusion and reporting of outcomes and analyses in systematic reviews of randomised trials of healthcare interventions. *The Cochrane database of systematic reviews* 2014(10):Mr000035. doi: https://doi.org/10.1002/14651858.MR000035.pub2 [published Online First: 2014/10/02]

17. Simera I, Moher D, Hirst A, et al. Transparent and accurate reporting increases reliability, utility, and impact of your research: reporting guidelines and the EQUATOR Network. *BMC Med* 2010;8:24. doi: https://doi.org/10.1186/1741-7015-8-24

18. Pollan M, Perez-Gomez B, Pastor-Barriuso R, et al. Prevalence of SARS-CoV-2 in Spain (ENE-COVID): a nationwide, population-based seroepidemiological study. *Lancet* 2020;396(10250):535-44. doi: 10.1016/S0140-6736(20)31483-5 [published Online First: 2020/07/10]

19. Bendavid E, Mulaney B, Sood N, et al. COVID-19 antibody seroprevalence in Santa Clara County, California. *International Journal of Epidemiology* 2021;50(2):410-19. doi: https://doi.org/10.1093/ije/dyab010

20. Abir T, Kalimullah NA, Osuagwu UL, et al. Prevalence and Factors Associated with Mental Health Impact of COVID-19 Pandemic in Bangladesh: A Survey-Based Cross-Sectional Study. *Ann Glob Health* 2021;87(1):43. doi: http://doi.org/10.5334/aogh.3269

21. Catalogue of Bias Collaboration. Catalogue of Bias 2017 [cited 2020. Available from: http://www.catalogofbias.org accessed 24 March 2022.

22. Riley S, Wang H, Eales O, et al. REACT-1 round 12 report: resurgence of SARS-CoV-2 infections in England associated with increased frequency of the Delta variant. *medRxiv* 2021:2021.06.17.21259103. doi: https://doi.org/10.1101/2021.06.17.21259103

23. Brown CS, Clare K, Chand M, et al. Snapshot PCR surveillance for SARS-CoV-2 in hospital staff in England. *Journal of Infection* 2020;81(3):427-34. doi: https://doi.org/10.1016/j.jinf.2020.06.069

24. Groves RM, Peytcheva E. The Impact of Nonresponse Rates on Nonresponse Bias: A Meta-Analysis. *The Public Opinion Quarterly* 2008;72(2):167-89.

25. Redmond SM, Alexander-Kisslig K, Woodhall SC, et al. Genital Chlamydia prevalence in europe and non-European high income countries: systematic review and meta-analysis. *PLoS One* 2015;10(1):e0115753. doi: 10.1371/journal.pone.0115753

26. Lord SJ, Staub LP, Bossuyt PMM, et al. Target practice: choosing target conditions for test accuracy studies that are relevant to clinical practice. *BMJ* 2011;343:d4684. doi: https://doi.org/10.1136/bmj.d4684

27. Jeong HW, Kim S-M, Kim H-S, et al. Viable SARS-CoV-2 in various specimens from COVID-19 patients. *Clinical Microbiology and Infection* 2020;26(11):1520-24. doi: https://doi.org/10.1016/j.cmi.2020.07.020

For peer review only - http://bmjopen.bmj.com/site/about/guidelines.xhtml

28. Niedrig M, Patel P, El Wahed AA, et al. Find the right sample: A study on the versatility of saliva and urine samples for the diagnosis of emerging viruses. *BMC Infectious Diseases* 2018;18(1):707. doi: https://doi.org/10.1186/s12879-018-3611-x

29. Leeflang MMG, Bossuyt PMM, Irwig L. Diagnostic test accuracy may vary with prevalence: implications for evidence-based diagnosis. *Journal of Clinical Epidemiology* 2009;62(1):5-12. doi: 10.1016/j.jclinepi.2008.04.007

30. Buitrago-Garcia D, Ipekci AM, Heron L, et al. Occurrence and transmission potential of asymptomatic and presymptomatic SARS-CoV-2 infections: Update of a living systematic review and meta-analysis. *PLOS Medicine* 2022;19(5):e1003987. doi: 10.1371/journal.pmed.1003987

31. Kimball A, Hatfield K, Arons M, et al. Asymptomatic and Presymptomatic SARS-CoV-2 Infections in Residents of a Long-Term Care Skilled Nursing Facility — King County, Washington, March 2020. *MMWR Morbidity and Mortality Weekly Report* 2020;69 doi: http://dx.doi.org/10.15585/mmwr.mm6913e1

32. Lin YH, Chen CY, Wu SI. Efficiency and Quality of Data Collection Among Public Mental Health Surveys Conducted During the COVID-19 Pandemic: Systematic Review. *J Med Internet Res* 2021;23(2):e25118. doi: 10.2196/25118 [published Online First: 20210210]

33. Yousaf AR, Duca LM, Chu V, et al. A prospective cohort study in non-hospitalized household contacts with SARS-CoV-2 infection: symptom profiles and symptom change over time. *Clin Infect Dis* 2020 doi: 10.1093/cid/ciaa1072 [published Online First: 2020/07/29]

34. Daniels CJ, Rajpal S, Greenshields JT, et al. Prevalence of Clinical and Subclinical Myocarditis in Competitive Athletes With Recent SARS-CoV-2 Infection: Results From the Big Ten COVID-19 Cardiac Registry. *JAMA cardiology* 2021 doi: https://doi:10.1001/jamacardio.2021.2065 [published Online First: 2021/05/28]

35. Vandenbroucke JP, von Elm E, Altman DG, et al. Strengthening the Reporting of Observational Studies in Epidemiology (STROBE): explanation and elaboration. *International journal of surgery (London, England)* 2014;12(12):1500-24. doi: https://doi.org/10.1016/j.ijsu.2014.07.014 [published Online First: 2014/07/22]

36. Galea S, Tracy M. Participation rates in epidemiologic studies. *Ann Epidemiol* 2007;17(9):643-53. doi: 10.1016/j.annepidem.2007.03.013 [published Online First: 2007/06/08]

37. Groves RM. Nonresponse Rates and Nonresponse Bias in Household Surveys. *The Public Opinion Quarterly* 2006;70(5):646-75.

38. Baumann L, Cina M, Egli-Gany D, et al. Prevalence of Mycoplasma genitalium in different population groups: systematic review andmeta-analysis. *Sex Transm Infect* 2018;94(4):255-62. doi: https://doi:10.1136/sextrans-2017-053384

39. Chu DKW, Pan Y, Cheng SMS, et al. Molecular Diagnosis of a Novel Coronavirus (2019-nCoV) Causing an Outbreak of Pneumonia. *Clinical chemistry* 2020;66(4):549-55. doi: https://doi.org/10.1093/clinchem/hvaa029 [published Online First: 2020/02/08]

40. Corman VM, Landt O, Kaiser M, et al. Detection of 2019 novel coronavirus (2019-nCoV) by real-time RT-PCR. *Eurosurveillance* 2020;25(3):2000045. doi: https://doi.org/10.2807/1560-7917.ES.2020.25.3.2000045

For peer review only

For peer review only - http://bmjopen.bmj.com/site/about/guidelines.xhtml

41. Whitaker M, Elliott J, Chadeau-Hyam M, et al. Persistent symptoms following SARS-CoV-2 infection in a random community sample of 508,707 people. *medRxiv* 2021:2021.06.28.21259452. doi: https://doi.org/10.1101/2021.06.28.21259452

42. Michelen M, Manoharan L, Elkheir N, et al. Characterising long COVID: a living systematic review. *BMJ Glob Health* 2021;6(9) doi: 10.1136/bmjgh-2021-005427

43. World Health Organization. A clinical case definition of post COVID-19 condition by a Delphi consensus 2021 [updated 6 October 2021. Available from: https://www.who.int/publications/i/item/WHO-2019-nCoV-Post_COVID-19_condition-Clinical_case_definition-2021.1.

44. Raynaud M, Zhang H, Louis K, et al. COVID-19-related medical research: a meta-research and critical appraisal. *BMC medical research methodology* 2021;21(1):1. doi: 10.1186/s12874-020-01190-w

45. Ipekci AM, Buitrago-Garcia D, Meili KW, et al. Outbreaks of publications about emerging infectious diseases: the case of SARS-CoV-2 and Zika virus. *BMC Med Res Methodol* 2021;21(1):50. doi: 10.1186/s12874-021-01244-7 [published Online First: 2021/03/13]

Declarations

Funding: This work received support from the Swiss government excellence scholarship (Grant number 2019.0774), the SSPH+ Global PhD Fellowship Programme in Public Health Sciences of the Swiss School of Public Health, and the Swiss National Science Foundation (project number 176233, and National Research Programme 78 COVID-19, project number 198418).

Authors' contributions: Diana Buitrago-Garcia, Georgia Salanti and Nicola Low conceptualised the project. Diana Buitrago-Garcia and Nicola Low wrote the manuscript. Georgia Salanti and Nicola Low provided feedback. Georgia Salanti and Nicola Low supervised the research. All authors edited the manuscript and approved the final manuscript.

Ethics approval: Not applicable

Consent to participate: Not applicable

Consent for publication: Not applicable

For peer review only - http://bmjopen.bmj.com/site/about/guidelines.xhtml

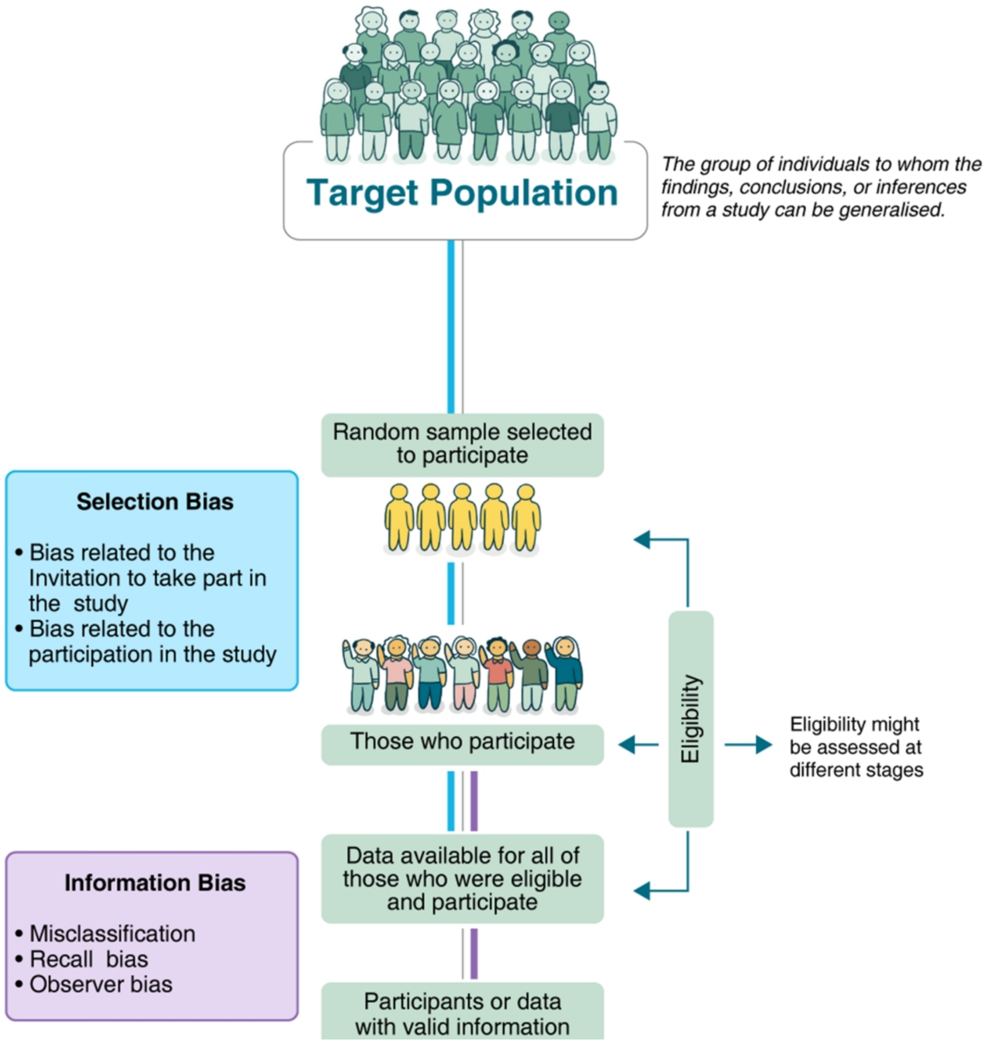

Potential for selection and information bias in prevalence studies. Coloured lines relate to the coloured boxes, showing at which stage of study procedures selection bias (blue line) and information bias (purple line) can occur.

433x458mm (118 x 118 DPI)

BMJ Open

Studies of prevalence. How a basic epidemiology concept has gained recognition in the COVID-19 pandemic.

Journal:	*BMJ Open*
Manuscript ID	bmjopen-2022-061497.R3
Article Type:	Communication
Date Submitted by the Author:	24-Sep-2022
Complete List of Authors:	Buitrago-Garcia, Diana; University of Bern Faculty of Medicine, Institute of Social and Preventive Medicine (ISPM); University of Bern, Graduate School of Health Sciences<br>Salanti, Georgia; University of Bern Faculty of Medicine, Institute of Social and Preventive Medicine<br>Low, Nicola; University of Bern Faculty of Medicine, Institute of Social and Preventive Medicine
**Primary Subject Heading**:	Epidemiology
Secondary Subject Heading:	Infectious diseases
Keywords:	EPIDEMIOLOGY, COVID-19, STATISTICS & RESEARCH METHODS

SCHOLARONE™
Manuscripts

I, the Submitting Author has the right to grant and does grant on behalf of all authors of the Work (as defined in the below author licence), an exclusive licence and/or a non-exclusive licence for contributions from authors who are: i) UK Crown employees; ii) where BMJ has agreed a CC-BY licence shall apply, and/or iii) in accordance with the terms applicable for US Federal Government officers or employees acting as part of their official duties; on a worldwide, perpetual, irrevocable, royalty-free basis to BMJ Publishing Group Ltd ("BMJ") its licensees and where the relevant Journal is co-owned by BMJ to the co-owners of the Journal, to publish the Work in BMJ Open and any other BMJ products and to exploit all rights, as set out in our licence.

The Submitting Author accepts and understands that any supply made under these terms is made by BMJ to the Submitting Author unless you are acting as an employee on behalf of your employer or a postgraduate student of an affiliated institution which is paying any applicable article publishing charge ("APC") for Open Access articles. Where the Submitting Author wishes to make the Work available on an Open Access basis (and intends to pay the relevant APC), the terms of reuse of such Open Access shall be governed by a Creative Commons licence – details of these licences and which Creative Commons licence will apply to this Work are set out in our licence referred to above.

Other than as permitted in any relevant BMJ Author's Self Archiving Policies, I confirm this Work has not been accepted for publication elsewhere, is not being considered for publication elsewhere and does not duplicate material already published. I confirm all authors consent to publication of this Work and authorise the granting of this licence.

For peer review only

Studies of prevalence. How a basic epidemiology concept has gained recognition in the COVID-19 pandemic

Diana Buitrago-Garcia

Institute of Social and Preventive Medicine, Graduate School of Health Sciences, University of Bern, Bern, Switzerland (0000-0001-9761-206X)

Email: diana.buitragogarcia@ispm.unibe.ch

Georgia Salanti

Institute of Social and Preventive Medicine, University of Bern, Bern, Switzerland (0000-0002-3830-8508)

Email: georgia.salanti@ispm.unibe.ch

Nicola Low

Institute of Social and Preventive Medicine, University of Bern, Bern, Switzerland (0000-0003-4817-8986)

Email: nicola.low@ispm.unibe.ch

Keywords

Prevalence, Observational Study, Bias, Selection Bias, Epidemiology, COVID-19

Main text 2849 words, 1 figure, 1 table

For peer review only

For peer review only - http://bmjopen.bmj.com/site/about/guidelines.xhtml

Abstract

Prevalence measures the occurrence of any health condition, exposure, or other factors related to health. The experience of COVID-19, a new disease caused by severe acute respiratory syndrome coronavirus 2 (SARS-CoV-2), has highlighted the importance of prevalence studies, for which issues of reporting and methodology have traditionally been neglected. This commentary highlights key issues about risks of bias in the design and conduct of prevalence studies and in reporting them, using examples about SARS-CoV-2 and COVID-19. The two main domains of bias in prevalence studies are those related to the study population (selection bias) and the condition or risk factor being assessed (information biases). Sources of selection bias should be considered both at the time of the invitation to take part in a study and when assessing who participates and provides valid data (respondents and non-respondents). Information bias appears when there are systematic errors affecting the accuracy and reproducibility of the measurement of the condition or risk factor. Types of information bias include misclassification, observer and recall bias. When reporting prevalence studies, clear descriptions of the target population, study population, study setting and context, and clear definitions of the condition or risk factor and its measurement, are essential. Without clear reporting, the risks of bias cannot be assessed properly. Bias in the findings of prevalence studies can, however, impact decision-making and the spread of disease. The concepts discussed here can be applied to the assessment of prevalence for many other conditions. Efforts to strengthen methodological research and improve assessment of the risk of bias and the quality of reporting of studies of prevalence in all fields of research should continue beyond this pandemic.

For peer review only - http://bmjopen.bmj.com/site/about/guidelines.xhtml

In introductory epidemiology, students learn about prevalence, an easy to understand concept, defined as "*a proportion that measures disease occurrence of any type of health condition, exposure, or other factor related to health*",[1] or "*the proportion of persons in a population who have a particular disease or attribute at a specified point in time or over a specified period.*"[2] Prevalence is an important measure for assessing the magnitude of health-related conditions, and studies of prevalence are an important source of information for estimating the burden of disease, injuries and risk factors.[3] Accurate information about prevalence enables health authorities to assess the health needs of a population, to develop prevention programmes and prioritise resources to improve public health.[4] Perhaps, owing to the apparent simplicity of the concept of prevalence, methodological developments to assess the quality of reporting, the potential for bias, and the synthesis of prevalence estimates in meta-analysis have been neglected,[5] when compared with the attention paid to methods relevant to evidence from randomised controlled trials and comparative observational studies.[6 7]

The COVID-19 pandemic has shown the need for epidemiological studies to describe and understand a new disease quickly but accurately.[8] Studies reporting on prevalence have been an important source of evidence to describe the prevalence of active SARS-CoV-2 infection and antibodies to SARS-CoV-2, the spectrum of SARS-CoV-2-related morbidity and helped to understand factors related to infection and disease to inform national decisions about containment measures.[9-11] Accurate estimates of prevalence of SARS-CoV-2 are crucial because they are used as an input for the estimation of other quantities, such as infection fatality ratios, which can be calculated indirectly using seroprevalence estimates.[12] Assessments of published studies have, however, highlighted methodological issues that affect study design, conduct, analysis, interpretation, and reporting.[13-15] In addition, some questions about prevalence need to be addressed through systematic reviews and meta-epidemiological studies. A high proportion of published systematic reviews of prevalence, however, also have flaws in reporting and methodological quality.[5 16] Confidence in the results of systematic reviews is determined by the credibility of the primary studies and the methods used to synthesise them.

The objective of this communication is to highlight key issues about the risk of bias in studies that measure prevalence and about the quality of reporting, using examples about SARS-CoV-2 and

For peer review only - http://bmjopen.bmj.com/site/about/guidelines.xhtml

COVID-19. We refer to prevalence at the level of a population, and not as a prediction at an individual level. The estimand is therefore "what proportion of the population is positive" and not "what is the probability this person is positive." Although incidence and prevalence are related epidemiologically, we do not discuss incidence in this article because the study designs for measurement of the quantities differ. Bias is a systematic deviation of results or inferences from the underlying (unobserved) true values.[1] The risk of bias is a judgment about the degree to which the methods or findings of a study might under-or over-estimate the true value in the target population,[7] in this case, the prevalence of a condition or risk factor. Quality of reporting refers to the completeness and transparency of the presentation of a research publication.[17] Risk of bias and quality of reporting are separate, but closely related, because it is only possible to assess the strengths and weaknesses of a study report if the methods and results are described adequately.

Bias in prevalence studies

The two main domains of bias in prevalence studies are those related to the study population (selection bias) and the condition being assessed (information biases) (Fig. 1). Biases involved in the design, conduct and analysis of a study affect its internal validity. Selection bias also affects external validity, the extent to which findings from a specific study can be generalised to a wider, target population in time and space. There are many names given to different biases, often addressing the same concept. For this communication, we use the names and definitions published in the Dictionary of Epidemiology.[1]

Selection bias

Selection bias relates to the representativeness of the sample used to estimate the prevalence in relation to the target population. The target population is the group of individuals to whom the findings, conclusions, or inferences from a study can be generalised.[1] There are two steps in a prevalence study at which selection bias might occur: at the invitation to take part in the study and, among those invited, who take part (Fig. 1).

[Fig 1.]

For peer review only

Selection bias in the invitation to take part in the study: The probability of being invited to take part in a study should be the same for every person in the target population. Evaluation of selection bias at this stage should therefore account for the complexity of the strategy for identification of participants. For example, if participants are invited from people who have previously agreed to participate in a registry or cohort, each level of invitation that has contributed to the final setting should be judged for the increasing risk of self-selection. Those who are invited to take part might be defined by demographic characteristics, e.g., children below ten years or study setting (e.g., hospitalised patients), or a random sample of the general population. The least biased method to select participants in a prevalence study is to sample at random from the target population. For example, the Real-time Assessment of Community Transmission (REACT) Studies to assess the prevalence of the virus, using molecular diagnostic tests (REACT-1) and antibodies (REACT-2), invite random samples of people, stratified by area, from the National Health Service patient list in England.[9] Those invited are close to a truly random sample because almost everyone in England is registered with a general practitioner. In some cases, criteria applied to the selection of a random sample might still result in considerable bias. For example, a seroprevalence study conducted in Spain did not include care home addresses, which could have excluded around 6% of the Spanish older population.[18] Excluding people in care homes facilities might underestimate SARS-CoV-2 seroprevalence in older adults, if their risk of exposure was higher than the average in the general population.[13] Other methods of sampling are at risk of selection bias. For example, asking for volunteers through advertisements are liable to selection bias because not everyone has the same probability of seeing or replying to the advert. For example, the use of social media to invite people to a drive-in test centre to estimate the population prevalence of antibodies to SARS-CoV-2,[19] or online adverts to assess mental health symptoms during the pandemic, excludes those without an internet connection or who do not use social media, such as older people.[20]

Selection bias related to who takes part in the study: Non-response bias occurs when people who have been invited, but do not take part in a study differ systematically from those who take part in ways that are associated with the condition of interest.[21] In the REACT-1 study,[22] for example, across four survey rounds, the investigators invited 2.4 million people; 596,000 swabs that were returned had a

For peer review only

valid result (25%). The proportion of participants responding was lower in later than in earlier rounds, in men than women and in younger than older age groups. If the sociodemographic characteristics of the target population are known, the observed results could be weighted statistically to represent the overall population but might still be biased by unmeasurable characteristics that drive willingness to take part.

The direction of non-response bias is often not predictable (can result in over-or underestimation of the true prevalence) because information about the motivation to take part in a study, or not, is not usually collected.[13] In a multicentre cross-sectional survey of the prevalence of PCR-determined SARS-CoV-2 in hospitals in England, the authors suggested that different selection biases could have had opposing effects.[23] For example, staff might have volunteered to take part if they were concerned that they might have been exposed to COVID-19. If such people were more likely than unexposed people to be tested, prevalence might be over-estimated. Alternatively, workers in lower-paid jobs, without financial support might have been less likely to take part than those at higher grades because of the consequences for themselves or their contacts if found to be infected. If the less-well paid jobs are also associated with a higher risk of exposure to SARS-CoV-2, the prevalence in the study population would be underestimated. Accorsi et al. suggest that the risk of non-response bias in seroprevalence studies might be reduced by sampling from established and well-characterised cohorts with high levels of participation, in whom the characteristics of non-respondents are known.[13]

As the proportion of invited people that do not take part in a study (non-respondents) increases, the probability of non-response bias might also increase if the topic of the study influences the probability and the composition of the study populationy.[24] Empirical evidence of bias was found in a systematic review of sexually transmitted *Chlamydia trachomatis* infection; prevalence surveys with the lowest proportion of respondents found the highest prevalence of infection, suggesting selective participation by those with a high risk of being infected.[25] Whether or not there is a dose-response relationship between the proportion of non-respondents and the likelihood of SARS-CoV-2 infection is unclear. The risks of selection bias at the stages of invitation and participation can be interrelated and might oppose each other. In the REACT-1 study,[22] it is not clear whether the reduction in selection bias

For peer review only

For peer review only - http://bmjopen.bmj.com/site/about/guidelines.xhtml

through random sampling outweighed the potential for selection bias owing to the high and increasing proportion of non-respondents over time, or vice versa.

Information bias

Information bias occurs when there are systematic errors affecting the completeness or accuracy of the measurement of the condition or risk factor of interest. There are different types of information bias.

Misclassification bias: This refers to the incorrect classification of a participant as having, or not having, the condition of interest. Misclassification is an important source of measurement bias in prevalence studies because diagnostic tests are imperfect and might not distinguish clearly among those with and without the condition.[26] For diagnostic tests, the predictive values will also be influenced by the prevalence of the condition in the study population. Seroprevalence studies are essential for determining the proportion of a population that has been exposed to SARS-CoV-2 up to a given time point. Detection of antibodies is affected by the test type and manufacturer, sample type such as serum, dried blood spots, saliva, urine or others,[27 28] and the time of sampling after infection. Different diagnostic tests might also be used in participants in the same study population, but adjustment for test performance is not always appropriate because the characteristics derived from studies in which the tests were validated might differ from the study population.[13] Accorsi et al. have described in detail this issue and other biases in the ascertainment of SARS-CoV-2 seroprevalence studies.[13] Test accuracy can also change across populations, owing to the inherent characteristics of tests when clinical variability is present,[29] for example when tests for SARS-CoV-2 detection are applied to people with or without symptoms.

In a new disease, such as COVID-19, diagnostic criteria might not be standardised or might change over time. For example, accurate assessment of the prevalence of persistent asymptomatic SARS-CoV-2 infection requires a complete list of symptoms and follow-up for a sufficiently long duration to ensure that symptoms did not develop later.[15 30] In a prevalence study conducted in a care home in March 2020, patients were asked about typical and non-typical symptoms of COVID-19. However, symptoms such as anosmia or ageusia had not been reported in association with SARS-CoV-2 at that time, so patients with these as isolated symptoms could have been wrongly classified as

For peer review only

asymptomatic.[15 31] Poor quality of data collection has also been found in studies estimating the prevalence of mental health problems during the pandemic.[32] The use of non-validated scales, or dichotomisation to define the cases using inappropriate or unclear thresholds, will bias the estimated prevalence of the condition. Misclassification may also occur in calculations of the prevalence of SARS-CoV-2 in contacts of diagnosed cases if not all contacts are tested, and it is assumed that individuals that were not tested were also uninfected.[13]

Recall bias: this bias results in misclassification when the condition has been measured through surveys or questionnaires that rely on memory. A study that aimed to describe the characteristics and symptom profile of individuals with SARS-CoV-2 infection in the United States collected information about symptoms before, and for 14 days after, being enrolled in the study.[33] The authors discuss the potential for recall bias when collecting symptoms retrospectively and if different people recollect different symptoms.

Observer bias: This bias occurs when an observer provides a wrong measurement due to lack of training or subjectivity.[21] For example, a study in the United States of America found variation between 14 universities in the prevalence of clinical and subclinical myocarditis in competitive athletes with SARS-CoV-2 infection.[34] One of the diagnostic tools was cardiac magnetic resonance and authors attributed some of the variability to differences in the protocols and the expertise among assessors. To reduce the risk of observer bias, researchers should aim to use tools that minimise subjectivity and standardise training procedures.

Reporting studies of prevalence

There is no agreed list of preferred items for reporting studies of prevalence. The published article or a preprint are usually the only available record of a study to which most people, other than the investigators themselves, have access. The written report therefore needs to contain the information required to understand the possible biases and assess internal and external validity. The Strengthening the Reporting of Observational Studies in Epidemiology (STROBE) statement is a widely used guideline, which includes recommendations for cross-sectional studies that examine associations between an exposure and outcome.[35] Table 1 shows selected items from the STROBE statement and

For peer review only

For peer review only - http://bmjopen.bmj.com/site/about/guidelines.xhtml

recommendations for cross-sectional studies that are particularly relevant to the complete and transparent description of methods for studies of prevalence.

Table 1. Items from the STROBE checklist for cross-sectional studies that are relevant for prevalence studies

Item	Item number	Recommendation
Setting	5	Describe the setting, locations, and relevant dates, including periods of recruitment, exposure, follow-up, and data collection
Participants	6a	Give the eligibility criteria, and the sources and methods of selection of participants
Variables	7	Clearly define all outcomes, exposures, predictors, potential confounders, and effect modifiers. Give diagnostic criteria, if applicable
Data sources/ measurement	8	For each variable of interest, give sources of data and details of methods of assessment (measurement). Describe comparability of assessment methods if there is more than one group.
Statistical methods	12c	Explain how missing data were addressed
	12d	If applicable, describe analytical methods taking account of sampling strategy
Participants	13a	Report numbers of individuals at each stage of study—eg numbers potentially eligible, examined for eligibility, confirmed eligible, included in the study, completing follow-up, and analysed
	13b	Give reasons for non-participation at each stage

First, clear definitions of the target population, study setting and eligibility criteria to select the study population are required (STROBE items 5, 6a). These issues affect assessment of external validity[36] because estimates of prevalence in a specific population and setting are often generalised more widely.[1 14] Second, the denominator used to calculate the prevalence should be clearly stated, with a

For peer review only - http://bmjopen.bmj.com/site/about/guidelines.xhtml

description of each stage of the study showing the numbers of individuals eligible, included, and analysed (STROBE item 13a, b). Accurate reports of the numbers and characteristics of those who take part (responders) or do not take part (non-responders) in the study are needed for the assessment of selection bias, but this information is not always available.[24 37] Poor reporting about the proportion of responders has been described as one of the main limitations of studies in systematic reviews of prevalence.[38] As with reports of studies of any design, the statistical methods applied to provide prevalence estimates, including methods used to address missing data (STROBE item 12c) and to account for the sampling strategy (STROBE item 12d) need to be reported clearly.[35] The setting, location and periods of enrolment and data collection (STROBE item 5) are particularly important for studies of SARS-CoV-2; the stage of the pandemic, preventive measures in place and virus variants in circulation should all be described because these affect the interpretation of estimates of prevalence. Third, it is crucial to provide a clear definition of the condition or risk factor of interest (STROBE item 7) and how it was measured (STROBE item 8), so that the risk of information bias can be assessed. The definition may be straightforward if there are objective criteria for ascertainment. For example, studies of the prevalence of active SARS-CoV-2 infection should report the diagnostic test, manufacturer, sample type, and criteria for a positive result.[39 40] For new conditions that have not been fully characterised, such as post COVID-19 condition, also known as 'long COVID', reporting of prevalence can be challenging.[41 42] The World Health Organization produced a case definition[43] in October 2021, but this might take time to be adopted widely.

Conclusion

The COVID-19 pandemic has produced an enormous amount of research about a single disease, published over a short time period.[44 45] Authors who have assessed the body of research on COVID-19 have highlighted concerns about the risks of bias in different study designs, including studies of prevalence.[13 44] In systematic reviews of a single topic, the occurrence of asymptomatic SARS-CoV-2 infection, we observed high between-study heterogeneity, serious risks of bias and poor reporting in the measurement of prevalence.[30] Biased results from prevalence studies can have a direct impact at the levels of the individual, community, global health, and policymaking. This communication

For peer review only - http://bmjopen.bmj.com/site/about/guidelines.xhtml

For peer review only

describes concepts about risks of bias and provides examples that authors can apply to the assessment of prevalence for many other conditions. Future research should be conducted to investigate sources of bias in studies of prevalence and empirical evidence of their influence on estimates of prevalence. The development of a tool that can be adapted to assess the risk of bias in studies of prevalence, and an extension to the STROBE reporting guideline, specifically for studies of prevalence, would help to improve the quality of published studies of prevalence in all fields of research beyond this pandemic.

Figure legend

Potential for selection bias in prevalence studies. Coloured lines relate to the coloured boxes, showing at which stage of study procedures selection bias (blue line) and information bias (purple line) can occur.

For peer review only

For peer review only - http://bmjopen.bmj.com/site/about/guidelines.xhtml

References

1. Porta M. A Dictionary of Epidemiology Oxford, United States: Oxford University Press; 2014 [6th Edition:[Available from: https://www.oxfordreference.com/view/10.1093/acref/9780195314496.001.0001/acref-9780195314496. accessed 24 March 2022.

2. Rothman KJ, Greenland S, Lash TL. Measures of Occurrence In: Rothman KJ, Greenland S, eds. Modern Epidemiology. Boston, Massachusetts: Lippincott Williams & Wilkins 2020:32-50.

3. Institute for Health Metrics and Evaluation (IHME). The Global Burden of Disease Study: University of Washington; 2020 [Available from: http://www.healthdata.org/gbd/about accessed 24 March 2022.

4. Vos T, Lim SS, Abbafati C, et al. Global burden of 369 diseases and injuries in 204 countries and territories, 1990-2019: a systematic analysis for the Global Burden of Disease Study 2019. *Lancet* 2020;396(10258):1204-22. doi: https://doi.org/10.1016/S0140-6736(20)30925-9

5. Hoffmann F, Eggers D, Pieper D, et al. An observational study found large methodological heterogeneity in systematic reviews addressing prevalence and cumulative incidence. *J Clin Epidemiol* 2020;119:92-99. doi: https://doi.org/10.1016/j.jclinepi.2019.12.003

6. Egger M, Davey-Smith G, Altman D. Systematic Reviews in Health Care: Meta-Analysis in Context. 2nd ed: Wiley 2001.

7. Higgins JPT, Thomas J, Chandler J, et al. Cochrane Handbook for Systematic Reviews of Interventions Version 6.2 (updated February 2021): Cochrane; 2021 [Available from: www.training.cochrane.org/handbook. accessed 24 March 2022.

8. Lipsitch M, Swerdlow DL, Finelli L. Defining the Epidemiology of Covid-19 — Studies Needed. *New England Journal of Medicine* 2020;382(13):1194-96. doi: https://doi/full/10.1056/NEJMp2002125

9. Riley S, Atchison C, Ashby D, et al. REal-time Assessment of Community Transmission (REACT) of SARS-CoV-2 virus: Study protocol. *Wellcome Open Res* 2021;5:200. doi: https://doi.org/10.12688/wellcomeopenres.16228.2

10. World Health Organization. Population-based age-stratified seroepidemiological investigation protocol for coronavirus 2019 (COVID-19) infection. Version 2.0. Geneva, 2020.

11. Siegler AJ, Sullivan PS, Sanchez T, et al. Protocol for a national probability survey using home specimen collection methods to assess prevalence and incidence of SARS-CoV-2 infection and antibody response. *Annals of Epidemiology* 2020;49:50-60. doi: https://doi.org/10.1016/j.annepidem.2020.07.015

12. Levin AT, Hanage WP, Owusu-Boaitey N, et al. Assessing the age specificity of infection fatality rates for COVID-19: systematic review, meta-analysis, and public policy implications. *Eur J Epidemiol* 2020;35(12):1123-38. doi: 10.1007/s10654-020-00698-1 [published Online First: 2020/12/09]

13. Accorsi EK, Qiu X, Rumpler E, et al. How to detect and reduce potential sources of biases in studies of SARS-CoV-2 and COVID-19. *European journal of epidemiology* 2021:179-96. doi: https://doi.org/10.1007/s10654-021-00727-7 [published Online First: 2021/02/27]

For peer review only - http://bmjopen.bmj.com/site/about/guidelines.xhtml

14. Griffith GJ, Morris TT, Tudball MJ, et al. Collider bias undermines our understanding of COVID-19 disease risk and severity. *Nature Communications* 2020;11(1):5749. doi: https://doi.org/10.1038/s41467-020-19478-2

15. Meyerowitz EA, Richterman A, Bogoch I, et al. Towards an accurate and systematic characterisation of persistently asymptomatic infection with SARS-CoV-2. *The Lancet Infectious diseases* 2021;21(6):e163-e69. doi: https://doi.org/10.1016/S1473-3099(20)30837-9 [published Online First: 2020/12/11]

16. Page MJ, McKenzie JE, Kirkham J, et al. Bias due to selective inclusion and reporting of outcomes and analyses in systematic reviews of randomised trials of healthcare interventions. *The Cochrane database of systematic reviews* 2014(10):Mr000035. doi: https://doi.org/10.1002/14651858.MR000035.pub2 [published Online First: 2014/10/02]

17. Simera I, Moher D, Hirst A, et al. Transparent and accurate reporting increases reliability, utility, and impact of your research: reporting guidelines and the EQUATOR Network. *BMC Med* 2010;8:24. doi: https://doi.org/10.1186/1741-7015-8-24

18. Pollan M, Perez-Gomez B, Pastor-Barriuso R, et al. Prevalence of SARS-CoV-2 in Spain (ENE-COVID): a nationwide, population-based seroepidemiological study. *Lancet* 2020;396(10250):535-44. doi: 10.1016/S0140-6736(20)31483-5 [published Online First: 2020/07/10]

19. Bendavid E, Mulaney B, Sood N, et al. COVID-19 antibody seroprevalence in Santa Clara County, California. *International Journal of Epidemiology* 2021;50(2):410-19. doi: https://doi.org/10.1093/ije/dyab010

20. Abir T, Kalimullah NA, Osuagwu UL, et al. Prevalence and Factors Associated with Mental Health Impact of COVID-19 Pandemic in Bangladesh: A Survey-Based Cross-Sectional Study. *Ann Glob Health* 2021;87(1):43. doi: http://doi.org/10.5334/aogh.3269

21. Catalogue of Bias Collaboration. Catalogue of Bias 2017 [cited 2020. Available from: http://www.catalogofbias.org accessed 24 March 2022.

22. Riley S, Wang H, Eales O, et al. REACT-1 round 12 report: resurgence of SARS-CoV-2 infections in England associated with increased frequency of the Delta variant. *medRxiv* 2021:2021.06.17.21259103. doi: https://doi.org/10.1101/2021.06.17.21259103

23. Brown CS, Clare K, Chand M, et al. Snapshot PCR surveillance for SARS-CoV-2 in hospital staff in England. *Journal of Infection* 2020;81(3):427-34. doi: https://doi.org/10.1016/j.jinf.2020.06.069

24. Groves RM, Peytcheva E. The Impact of Nonresponse Rates on Nonresponse Bias: A Meta-Analysis. *The Public Opinion Quarterly* 2008;72(2):167-89.

25. Redmond SM, Alexander-Kisslig K, Woodhall SC, et al. Genital Chlamydia prevalence in europe and non-European high income countries: systematic review and meta-analysis. *PLoS One* 2015;10(1):e0115753. doi: 10.1371/journal.pone.0115753

26. Lord SJ, Staub LP, Bossuyt PMM, et al. Target practice: choosing target conditions for test accuracy studies that are relevant to clinical practice. *BMJ* 2011;343:d4684. doi: https://doi.org/10.1136/bmj.d4684

27. Jeong HW, Kim S-M, Kim H-S, et al. Viable SARS-CoV-2 in various specimens from COVID-19 patients. *Clinical Microbiology and Infection* 2020;26(11):1520-24. doi: https://doi.org/10.1016/j.cmi.2020.07.020

For peer review only

For peer review only - http://bmjopen.bmj.com/site/about/guidelines.xhtml

28. Niedrig M, Patel P, El Wahed AA, et al. Find the right sample: A study on the versatility of saliva and urine samples for the diagnosis of emerging viruses. *BMC Infectious Diseases* 2018;18(1):707. doi: https://doi.org/10.1186/s12879-018-3611-x

29. Leeflang MMG, Bossuyt PMM, Irwig L. Diagnostic test accuracy may vary with prevalence: implications for evidence-based diagnosis. *Journal of Clinical Epidemiology* 2009;62(1):5-12. doi: 10.1016/j.jclinepi.2008.04.007

30. Buitrago-Garcia D, Ipekci AM, Heron L, et al. Occurrence and transmission potential of asymptomatic and presymptomatic SARS-CoV-2 infections: Update of a living systematic review and meta-analysis. *PLOS Medicine* 2022;19(5):e1003987. doi: 10.1371/journal.pmed.1003987

31. Kimball A, Hatfield K, Arons M, et al. Asymptomatic and Presymptomatic SARS-CoV-2 Infections in Residents of a Long-Term Care Skilled Nursing Facility — King County, Washington, March 2020. *MMWR Morbidity and Mortality Weekly Report* 2020;69 doi: http://dx.doi.org/10.15585/mmwr.mm6913e1

32. Lin YH, Chen CY, Wu SI. Efficiency and Quality of Data Collection Among Public Mental Health Surveys Conducted During the COVID-19 Pandemic: Systematic Review. *J Med Internet Res* 2021;23(2):e25118. doi: 10.2196/25118 [published Online First: 20210210]

33. Yousaf AR, Duca LM, Chu V, et al. A prospective cohort study in non-hospitalized household contacts with SARS-CoV-2 infection: symptom profiles and symptom change over time. *Clin Infect Dis* 2020 doi: 10.1093/cid/ciaa1072 [published Online First: 2020/07/29]

34. Daniels CJ, Rajpal S, Greenshields JT, et al. Prevalence of Clinical and Subclinical Myocarditis in Competitive Athletes With Recent SARS-CoV-2 Infection: Results From the Big Ten COVID-19 Cardiac Registry. *JAMA cardiology* 2021 doi: https://doi:10.1001/jamacardio.2021.2065 [published Online First: 2021/05/28]

35. Vandenbroucke JP, von Elm E, Altman DG, et al. Strengthening the Reporting of Observational Studies in Epidemiology (STROBE): explanation and elaboration. *International journal of surgery (London, England)* 2014;12(12):1500-24. doi: https://doi.org/10.1016/j.ijsu.2014.07.014 [published Online First: 2014/07/22]

36. Galea S, Tracy M. Participation rates in epidemiologic studies. *Ann Epidemiol* 2007;17(9):643-53. doi: 10.1016/j.annepidem.2007.03.013 [published Online First: 2007/06/08]

37. Groves RM. Nonresponse Rates and Nonresponse Bias in Household Surveys. *The Public Opinion Quarterly* 2006;70(5):646-75.

38. Baumann L, Cina M, Egli-Gany D, et al. Prevalence of Mycoplasma genitalium in different population groups: systematic review andmeta-analysis. *Sex Transm Infect* 2018;94(4):255-62. doi: https://doi:10.1136/sextrans-2017-053384

39. Chu DKW, Pan Y, Cheng SMS, et al. Molecular Diagnosis of a Novel Coronavirus (2019-nCoV) Causing an Outbreak of Pneumonia. *Clinical chemistry* 2020;66(4):549-55. doi: https://doi.org/10.1093/clinchem/hvaa029 [published Online First: 2020/02/08]

40. Corman VM, Landt O, Kaiser M, et al. Detection of 2019 novel coronavirus (2019-nCoV) by real-time RT-PCR. *Eurosurveillance* 2020;25(3):2000045. doi: https://doi.org/10.2807/1560-7917.ES.2020.25.3.2000045

For peer review only

For peer review only - http://bmjopen.bmj.com/site/about/guidelines.xhtml

41. Whitaker M, Elliott J, Chadeau-Hyam M, et al. Persistent symptoms following SARS-CoV-2 infection in a random community sample of 508,707 people. *medRxiv* 2021:2021.06.28.21259452. doi: https://doi.org/10.1101/2021.06.28.21259452

42. Michelen M, Manoharan L, Elkheir N, et al. Characterising long COVID: a living systematic review. *BMJ Glob Health* 2021;6(9) doi: 10.1136/bmjgh-2021-005427

43. World Health Organization. A clinical case definition of post COVID-19 condition by a Delphi consensus 2021 [updated 6 October 2021. Available from: https://www.who.int/publications/i/item/WHO-2019-nCoV-Post_COVID-19_condition-Clinical_case_definition-2021.1.

44. Raynaud M, Zhang H, Louis K, et al. COVID-19-related medical research: a meta-research and critical appraisal. *BMC medical research methodology* 2021;21(1):1. doi: 10.1186/s12874-020-01190-w

45. Ipekci AM, Buitrago-Garcia D, Meili KW, et al. Outbreaks of publications about emerging infectious diseases: the case of SARS-CoV-2 and Zika virus. *BMC Med Res Methodol* 2021;21(1):50. doi: 10.1186/s12874-021-01244-7 [published Online First: 2021/03/13]

Declarations

Funding: This work received support from the Swiss government excellence scholarship (Grant number 2019.0774), the SSPH+ Global PhD Fellowship Programme in Public Health Sciences of the Swiss School of Public Health, and the Swiss National Science Foundation (project number 176233, and National Research Programme 78 COVID-19, project number 198418).

Authors' contributions: Diana Buitrago-Garcia, Georgia Salanti and Nicola Low conceptualised the project. Diana Buitrago-Garcia and Nicola Low wrote the manuscript. Georgia Salanti and Nicola Low provided feedback. Georgia Salanti and Nicola Low supervised the research. All authors edited the manuscript and approved the final manuscript.

Acknowledgements: We would like to thank Yuly Barón, who created Figure 1.

Ethics approval: Not applicable

Consent to participate: Not applicable

Consent for publication: Not applicable

Competing interest: None

For peer review only - http://bmjopen.bmj.com/site/about/guidelines.xhtml

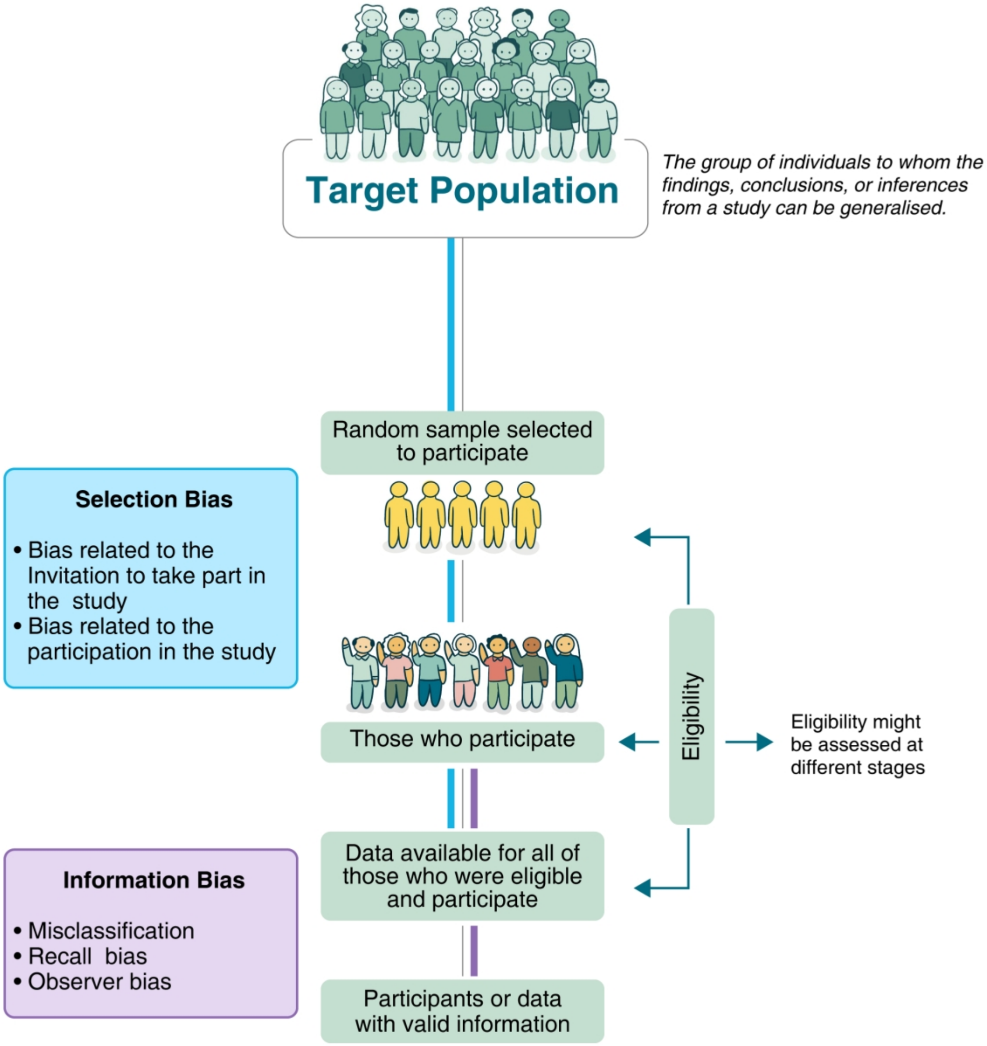

Potential for selection bias in prevalence studies. Coloured lines relate to the coloured boxes, showing at which stage of study procedures selection bias (blue line) and information bias (purple line) can occur.

170x183mm (300 x 300 DPI)
